# New Insights into the Catalytic Activity of Cobalt Orthophosphate Co_3_(PO_4_)_2_ from Charge Density Analysis

**DOI:** 10.1002/chem.201902303

**Published:** 2019-11-08

**Authors:** Helena Keil, Matti Hellström, Claudia Stückl, Regine Herbst‐Irmer, Jörg Behler, Dietmar Stalke

**Affiliations:** ^1^ Universität Göttingen Institut für Anorganische Chemie Tammannstrasse 4 37977 Göttingen Germany; ^2^ Universität Göttingen Institut für Physikalische Chemie, Theoretische Chemie Tammannstrasse 6 37077 Göttingen Germany; ^3^ Current address: Software for Chemistry and Materials B.V. De Boelelaan 1083 1081 HV Amsterdam The Netherlands

**Keywords:** charge density investigation, computational chemistry, cobalt phosphate, density of states, solid-state catalysis

## Abstract

An extensive characterization of Co_3_(PO_4_)_2_ was performed by topological analysis according to Bader‘s Quantum Theory of Atoms in Molecules from the experimentally and theoretically determined electron density. This study sheds light on the reactivity of cobalt orthophosphate as a solid‐state heterogeneous oxidative‐dehydration and ‐dehydrogenation catalyst. Various faces of the bulk catalyst were identified as possible reactive sites given their topological properties. The charge accumulations and depletions around the two independent five‐ and sixfold‐coordinated cobalt atoms, found in the topological analysis, are correlated to the orientation and population of the d‐orbitals. It is shown that the (011) face has the best structural features for catalysis. Fivefold‐coordinated ions in close proximity to advantageously oriented vacant coordination sites and electron depletions suit the oxygen lone pairs of the reactant, mainly for chemisorption. This is confirmed both from the multipole refinement as well as from density functional theory calculations. Nearby basic phosphate ions are readily available for C−H activation.

## Graphical abstract

**How to Cat**: The experimental and theoretical topological analysis of the electron density distribution in Co_3_(PO_4_)_2_ identified the (011) face to be best suited for catalysis. Fivefold‐coordinated Co ions in close proximity to advantageously oriented electron‐depletion sites suit the reactant's oxygen lone pairs most for chemisorption and heterogeneous C−H activation.

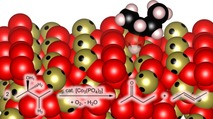

## Introduction

The synthesis of today's bulk chemicals heavily relies on catalysis, reducing energy consumption and preventing waste in atom‐economically tailored reaction sequences.^1^, ^2^ The design of appropriate reagents precisely adapted to the specific demand is one of the fundamental objectives in chemistry. Although the exact synthetic targets might differ, the aim is always the same: selective replacement of individual atoms or residues within a material to finetune its properties and by these means its reactivity or functionality. As a major drawback, it is still not always predictable which alterations cause the desired effects. Hence the benchmarking of working models along established concepts is required. The proper understanding of the structure–property relationship of a chemical system is vital for the improvement of the materials development process. Electron‐density (ED) investigations provide an ideal tool for understanding these interactions.^3^ Already in 1932, Pauling^4^ proposed an interrelation between the structure of a compound and its properties:

“The properties of a compound depend on two main factors, the nature of the bonds between the atoms, and the nature of the atomic arrangement. […] The satisfactory description of the atomic arrangement in a crystal or molecule necessitates the complete determination of the position of the atoms relative to one another.”

From the knowledge of the distances at the atomic level and the arrangement in the solid phase, many properties both at the molecular and macroscopic scale can be deduced. However, the most basic concepts, such as the chemical bond and reactivity, are still strongly discussable.

**Scheme 1:**
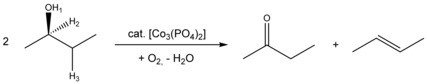
Catalytic reaction of butan‐2‐ol with oxygen to the products butene and butenone.^5^

Single‐crystal structural analyses based on the independent‐atom model only provide the positions of the centroids of the atoms and the distances between the atoms. In contrast, the multipole model from high‐resolution data provides physically meaningful properties far beyond simple geometrical considerations. In this paper we investigate the ED in catalytically active cobalt orthophosphate Co_3_(PO_4_)_2_ (Scheme 1) derived from both high‐resolution diffraction data and density functional theory (DFT) calculations, to get some insight in the various properties contributing to that activity. Not only the bonding is studied but also the ED distribution at the two different metal sites is determined and linked to various substrates.

## Results and Discussion

**Figure 1:**
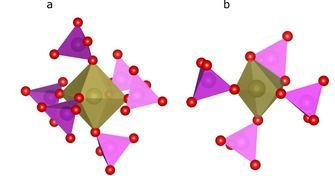
Different environments of a) Co^6^ and b) Co^[5by]^. The coordination polyhedron of phosphorus is colored in magenta and that of Co in bronze.

**Figure 2:**
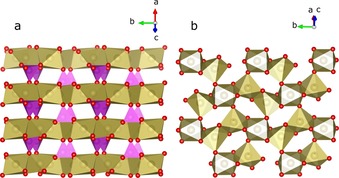
An excerpt from the crystal structure of cobalt phosphate, a) alternating layers of phosphates and cobalt atoms and b) connectivity of Co‐polyhedra. The coordination polyhedra of phosphorus are colored in magenta, those of Co are bronze. a, b, c are lattice constants.

Cobalt orthophosphate crystallizes in the monoclinic space group *P*2_1_/*n*, whereas in earlier publications^6^, ^7^ sometimes the other setting *P*2_1_/*c* was chosen. Furthermore, a metastable phase^8^ of cobalt orthophosphate is known in the nonstandard setting *P*2_1_/*b*. The unit cell contains two formula units of Co_3_(PO_4_)_2_. One of the two independent Co^II^ atoms is sixfold coordinated in an approximately ideal octahedral fashion (Figure 1 a). The second is fivefold coordinated, giving a severely distorted trigonal bipyramidal coordination polyhedron (Figure 1 b). The octahedrally coordinated Co ion is O‐corner‐sharing connected to six different phosphate ions; the Co inside the distorted bipyramidal polyhedron is only connected to four different anions (Figure 2). Three of these are corner‐sharing and one is edge‐sharing connected to the metal. The asymmetric unit contains only one half of the formula unit, hence the octahedrally coordinated Co atom is located on a symmetry center and the fivefold coordinated Co atom is on a general position. To judge whether the latter polyhedron is closer to a square‐pyramidal or to a trigonal‐bipyramidal structure, the τ parameter proposed by Addison^9^ was determined. It is defined as *τ*=(*β*−*α*)60° where *β* is the largest and *α* the second largest ligand‐metal‐ligand angle. For an ideal trigonal‐bipyramidal geometry, *τ* is unity and for an ideal square pyramidal geometry zero. Here, *τ* adopts a value of 0.67, which suggests a predominately trigonal‐bipyramidal environment.

Detailed bond lengths and angles are provided in the Supporting Information. For the sake of simplicity, the different Co atoms will from now‐on be termed Co^6^ and Co^[5by]^, respectively, according to Machatschki's terminology.^10^ Figure 2 a shows the single‐crystal structure composed from alternating layers of phosphate tetrahedra colored in magenta and Co polyhedra in bronze. The latter are connected through corners and edges to their adjacent Co polyhedra (Figure 2 b).

### Topological analysis

**Figure 3:**
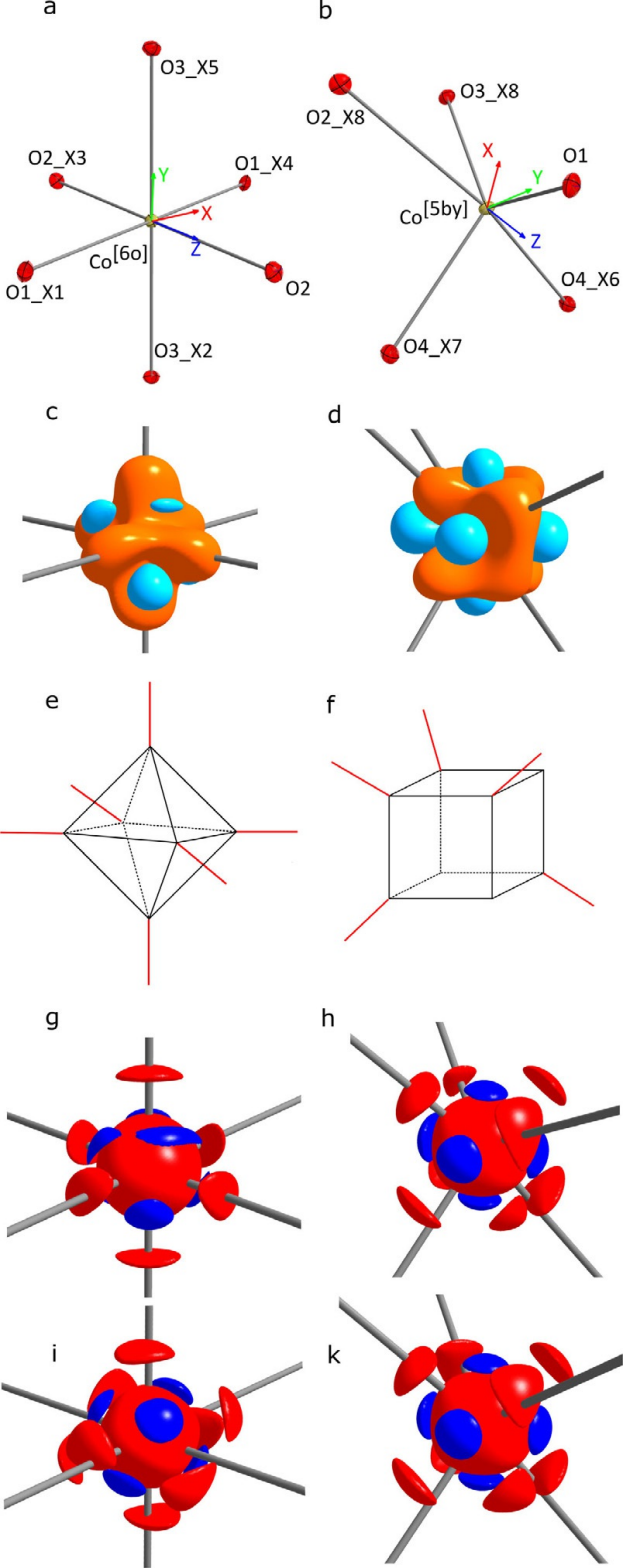
a) and b) local coordinate systems for Co^6^ and Co^[5by]^; c) and d) show the deformation density with the iso‐surface values of ±0.65 eÅ^−3^ for negative charge accumulation in light blue and positive charge in orange; e) and f) show the coordination sites; g) and h) depict the Laplacian with iso‐surfaces of 240 eÅ^−5^ (red) and −1250 eÅ^−5^ (dark blue) for the experimental, i) and k) theoretical data with iso‐surfaces of 260 eÅ^−5^ (red) and −1260 eÅ^−5^ (dark blue).

To model the ED more appropriately than feasible from the independent‐atom model (IAM), the multipole model (MM), based on the Hansen and Coppens formalism,^11^ was employed. It allows for the description of aspherical ED by spherical harmonics, in which the total density is partitioned in a spherical core density, a spherical valence, and an aspherical deformation valence density. To describe the aspherical density, local coordinate systems are necessary. We used DFT calculations, together with manual judgment, to determine the pertinent local coordinate systems, because the actual coordination polyhedra around the Co ions in Co_3_(PO_4_)_2_ are distorted from the ideal polyhedra (for details see the Supporting Information). The definition of the local coordinate systems is found in Figure 3 a and Figure 3 b. The thus derived ED can be investigated by a complete topological analysis according to QTAIM,^12^ based on the experimental diffraction data (MM‐Expt, MM=multipole model) as well as the structure factor derived from DFT calculations (MM‐DFT). In the Bader methodology, the presence of bond‐critical points (BCP) in the molecular graph is a prerequisite for an interaction between two atoms. The features at the BCP are also an indication for the bonding type and the strength of a bond, which might correlate with the distance.^12^ A consistent set of critical points was found for cobalt phosphate. They obey Morse's relation^13^ *n*−*b*+*r*−*c*=0, where *n*, *b*, *r*, and c are the numbers of nuclei, bond, ring, and cage‐critical points, respectively.

### Phosphate

Regarding the nature of the P−O bonds in phosphate, there is still a long‐standing discussion about the presence or absence of double‐bond character, hence also about the presence or absence of valence expansion at the phosphorus atom. Different theoretical calculations led to various binding models. Molina, Sundberg et al.^14^ investigated the bonding situation of several theoretically calculated and geometry optimized structures by means of QTAIM. They showed that the P−O bond in phosphane oxide is best described with a highly polar σ‐bond with a considerable electrostatic contribution. The same is valid for iminophosphoranes.^15^ Durrant^16^ calculated the number of valence electrons at the phosphorus atom in PS_4_^3−^ to be 7.94 and at the sulfur atom in sulfate to be 4.34. He, among many others, regarded the P−O bond in phosphate as very polar. For potassium sulfate, the high polarity of the S−O bond was already established in an experimental charge‐density investigation.^17^ In this study, we also show for phosphate that the bond between phosphorus and oxygen is a highly polar covalent bond with no double‐bond character.^18^ The evidence for this stems from the high ED and a positive Laplacian, the trace of the Hessian matrix of the second derivative of the ED function, at the BCP. As expected, for all four P−O bonds a high ED was found at the BCP, which suggests a covalent bond (Table 1). The values vary from 1.54 to 1.61 eÅ^−3^ and are in a good agreement with the theoretical data. For the Laplacian at the BCP, positive values were found in the range of 2–5 eÅ^−5^, which are slightly higher than those from theory (0–3 eÅ^−5^). Given that the BCP is located in a rampant edge of the Laplace function, even a small variation in the position of the BCP will cause a tremendous change in the numerical value and sometimes even a change of sign (Table 1, Figure S4‐2, Supporting Information). The ellipticity describes the deviation of the charge density around a bond path from an ideal cylindrical symmetry: *ϵ*=*λ*_1_ *λ*_2_−1, where *λ*_1_ and *λ*_2_ correspond to the eigenvectors of the Hessian matrix. Not surprisingly, we found *ϵ* values close to zero for all P−O bonds, corresponding to σ single bonds. Figure S5‐1 (Supporting Information) depicts the distances of the BCP from the oxygen‐atom position from theory (MM‐DFT) plotted above those from the experiment (MM‐Expt).

**Table 1:** Calculated BCPs and local energy density properties for MM‐Expt (bold) and MM‐DFT (normal). *R*: bond path; *ρ*(*r*_BCP_) ED at BCP; ∇^2^ *ρ*(*r*_BCP_): Laplacian at BCP; *ϵ*: ellipticity of the bond; *G*(*r*_BCP_): kinetic energy density at BCP; *V(r*_BCP_): potential energy density at BCP; *H*(*r*_BCP_): total energy density at BCP. The oxygen atoms marked with an X are generated by the symmetry code. X1: −1+*x*, +*y*, +*z*; X2: −1/2 +*x*, 1/2 −*y*, −1/2 +*z*; X3: −*x*, 1−*y*, 1−*z*; X4: 1−*x*, 1−*y*, 1−*z*; X5: 1/2 −*x*, 1/2 +*y*, $\frac{3}{2}$ −*z*; X6: $\frac{3}{2}$ −*x*, −1/2 +*y*, $\frac{3}{2}$ −*z*; X7: 1/2 +*x*, 1/2 −*y*, −1/2 +*z*; X8: 1+*x*, +*y*, +*z*. A picture of Co_3_(PO_4_)_2_ including these oxygen atoms can be found in the Supporting Information. For the sake of clarity, the values shown below have been rounded. The exact values for MM‐Expt and MM‐DFT including the standard deviation can be found in the Supporting Information.

A–B		*R*_(A‐B)_	*R*_(A‐BCP)_	*R*_(B‐BCP)_	*ρ*(*r*_BCP_)	∇^2^ *ρ*(*r*_BCP_)	*ϵ*	*G*(*r*_BCP_)/*ρ*(*r*_BCP_)	|*V*|(*r*_BCP_)	*H*(*r*_BCP_)/*ρ*(*r*_BCP_)
		[Å]	[Å]	[Å]	[eÅ^−3^]	[eÅ^−5^]		[E_h_/e]	/*G*(*r*_BCP_)	[E_h_/e]
Co^6^	O2	**2.0565**	**1.0081**	**1.0484**	**0.41**	**7.48**	**0.05**	**1.30**	**1.00**	**0.00**
		2.0563	1.0270	1.0294	0.43	7.32	0.06	1.25	1.06	−0.07
Co^6^	O1 X1	**2.1438**	**1.0464**	**1.0974**	**0.33**	**5.66**	**0.03**	**1.18**	**1.00**	**0.00**
		2.1437	1.0662	1.0776	0.36	5.66	0.07	1.14	1.02	−0.03
Co^6^	O3 X5	**2.1694**	**1.0555**	**1.1138**	**0.32**	**5.26**	**0.02**	**1.14**	**0.97**	**0.00**
		2.1694	1.0780	1.0914	0.34	5.17	0.05	1.10	1.05	−0.03
Co^5^	O1	**1.9774**	**0.9731**	**1.0043**	**0.52**	**9.94**	**0.05**	**1.41**	**1.05**	**−0.08**
		1.9772	0.9850	0.9922	0.53	9.60	0.03	1.37	1.08	−0.11
Co^5^	O2 X8	**2.2218**	**1.0942**	**1.1276**	**0.30**	**4.39**	**0.05**	**1.04**	**1.03**	**0.00**
		2.2217	1.1099	1.1118	0.30	4.31	0.07	1.03	1.03	−0.03
Co^5^	O3 X8	**2.0190**	**0.9995**	**1.0195**	**0.46**	**8.43**	**0.03**	**1.33**	**1.03**	**−0.07**
		2.0189	1.0094	1.0095	0.47	8.23	0.04	1.31	1.05	−0.06
Co^5^	O4 X6	**1.9889**	**0.9813**	**1.0076**	**0.51**	**9.64**	**0.04**	**1.40**	**1.04**	**−0.06**
		1.9888	0.9942	0.9946	0.52	9.36	0.02	1.36	1.07	−0.10
Co^5^	O4 X7	**2.0079**	**0.9937**	**1.0143**	**0.48**	**8.98**	**0.02**	**1.37**	**1.03**	**−0.06**
		2.0080	1.0033	1.0047	0.49	8.75	0.01	1.33	1.06	−0.08
P1	O1	**1.5334**	**0.6332**	**0.9002**	**1.59**	**4.82**	**0.03**	**1.24**	**1.83**	**−1.02**
		1.5333	0.6431	0.8902	1.58	2.59	0.01	1.17	1.90	−1.05
P1	O2	**1.5318**	**0.6327**	**0.8991**	**1.61**	**5.04**	**0.01**	**1.25**	**1.82**	**−1.02**
		1.5317	0.6423	0.8894	1.61	2.85	0.00	1.19	1.90	−1.06
P1	O3	**1.5556**	**0.6417**	**0.9139**	**1.54**	**1.93**	**0.02**	**1.13**	**1.93**	**−1.05**
		1.5556	0.6528	0.9028	1.54	−0.13	0.01	1.07	2.01	−1.07
P1	O4	**1.5413**	**0.6362**	**0.9051**	**1.57**	**3.70**	**0.01**	**1.20**	**1.86**	**−1.03**
		1.5414	0.6466	0.8948	1.57	1.51	0.01	1.13	1.94	−1.06

In the experiment, the BCP is slightly more shifted towards the electropositive phosphorus atom, indicating a marginally higher ionic character in the experimental than in DFT data. Cremer and Kraka^19^ proposed to use the total energy density *H* to classify bonds. For covalent bonds, the potential energy dominates, resulting in a negative total energy compared with closed shell interactions. In addition, as introduced by Espinosa et al.,^20^ the ratio of potential to kinetic‐energy density |V|G can be used to distinguish between shared, intermediate and closed‐shell interactions. For a closed‐shell interaction, the ratio |V|G at the bond‐critical point is less than unity, for a shared interaction greater than two, and for an intermediate interaction between one and two. The ratios |V|G for the P−O bonds ranging from 1.82 to 1.93 [1.90–2.01] are much more on the side of the shared interaction. If the ratio of the kinetic‐energy density per electron density at the BCP is greater than unity, this is indicative for polarity in a bond.

For the P−O bonds in phosphate, we found a *G*(*r*_bcp_)*ρ*(*r*_bcp_) ratio greater than unity (1.07 to 1.25). Maxima of the negative Laplacian represent Valence‐Shell Charge Contributions (VSCC). VSCCs in the nonbonding regions are indicative for lone pairs, frequently providing the link to the classical Lewis model and the VSEPR theory.^21^ For the phosphate oxygen atoms, four tetrahedrally oriented VSCCs would be expected due to the sp^3^ hybridization.^14^ However, the number of VSCCs could neither be resolved from MM‐Expt nor from MM‐DFT. As expected, one bonding VSCC is located near the P−O bond axis. The VSCCs in the nonbonding region pointing away from that bond are fused to a toroidal orientation. Even with full refinement of all feasible multipole parameters from theoretical data, it was impossible to resolve them. We refrained from that endeavor because it clearly indicated overfitting.^22^ Local mirror symmetry had to be taken into account. Additionally, the multipole population at all oxygen atoms had to be set to be the same.

### Co−O Polyhedra

Compared with the covalent P−O bonds, the Co−O bonds show a significantly lower ED in the range of approximately 0.32 to 0.52 eÅ^−3^ at the BCP. The Laplacian at the BCP is significantly more positive and ranges from 4 to 10 eÅ^−5^. The ED increase correlates with the reduction in distance. In the theoretical data set, *ρ*(*r*_BCP_) is slightly larger for all Co−O bonds, showing a larger diversity at the Co^6^−O bonds than the Co^[5by]^−O bonds. The found trend also reflects how the shared character increases with the decrease of the coordination number. Except for one, all Co^[5by]^−O bonds are shorter and have a greater value for *ρ*(*r*_BCP_) than the Co^6^−O bonds (Figure S5‐2, Supporting Information). This is also valid for many other M−O bonds. The value for ∇^2^ *ρ*(*r*_BCP_) increases with the decreasing bond distance, which has also been observed for other transition‐metal–oxygen interactions and nonmetal–oxygen interactions.^23^ Comparing the difference between the experimental and the theoretical data set, the values for ∇^2^ *ρ*(*r*_BCP_) in the Co−O bond are almost identical. The ratios |V|G for the Co−O bonds of 0.97–1.05 [1.02–1.08 from theory] indicate rather a closed‐shell interaction. A hint towards slightly shared interaction in the Co−O bond is the negative total energy density, *H* (*r*_BCP_).

### Bader charges

The Bader charges (Table S4‐4, Supporting Information) show for both, MM‐Expt and MM‐DFT, pronounced positive phosphorus (+3.94 e (MM‐Expt) and +3.69 e (MM‐DFT)) and cobalt atoms (+1.98 e (MM‐Expt) and +1.22 e (MM‐DFT) for Co^6^ and (+1.59 e (MM‐Expt) and +1.11 e (MM‐DFT) for Co^5^). As expected, the positive charge from both approaches is slightly higher for the six‐ than for the fivefold coordinated metal atom.

### Deformation density

The static deformation density is based on the difference between the total ED from the MM and a spherical reference ED from the IAM. This already provides a very informative picture of the ED distribution around the metal atoms. Regions of negative charge concentrations are depicted in light blue and those of negative charge depletion in orange (Figure 3 c and d). Interestingly, the number of charge‐concentration regions coincides in both Co^6^ and Co^[5by]^, but not the number of depleted regions. Co^6^ shows only six minima, each directed to the negatively charged oxygen atom of the phosphate, already fueling the idea of an electrostatic Co−O interaction. In contrast, Co^5^ shows eight regions of negative deformation density arranged at the eight corners of a cube. Five of the vertices point to a coordinated phosphate oxygen atom each and the remaining three exposed minima are left uncoordinated, indicating free coordination sites.

### Laplacian

The indications of the difference between the Co atoms found in the deformation density are echoed in the Laplacian. Each dark blue array in Figures 3 –h represents a VSCC (Table 2). The red regions represent the negative charge depletions. At Co^5^ the match between the features of the Laplacian from either the MM‐Expt and the MM‐DFT refinement is very good. At Co^6^, in contrast, differences such as the position and number of depletions are clear. The MM‐DFT data give eight negative charge‐depletion points arranged into an approximate cube (Figure 3 i), and six VSCCs roughly in the center of the faces formed by the cube of charge depletions.

**Table 2:** Experimental maxima of negative charge concentrations around the Co atoms. Listed are the values for the Laplacian, electron density and the distance of the maximum to the atom. The corresponding values for MM‐DFT are shown in the Supporting Information.

Atom	−∇^2^ *ρ* [eÅ^−5^]	*ρ* [eÅ^−3^]	d_VSCC‐atom_ [Å]
Co^6^	1367	29.24	0.3059
	1424	29.62	0.3054
	1737	30.96	0.3028
Co^5^	1793	31.53	0.3016
	1817	31.72	0.3014
	1862	32.07	0.3011
	1889	32.32	0.3009
	1890	32.29	0.3009
	1916	32.58	0.3008

The vertices of the charge‐depletion cube do not fully coincide with the ion–ligand bonds. In contrast, from the experimental multipole refinement (Figure 3 g), there are only six charge depletions, that form an octahedron with vertices approximately along the six ion–ligand bonds.

### Density of states and d‐orbital population

**Figure 4:**
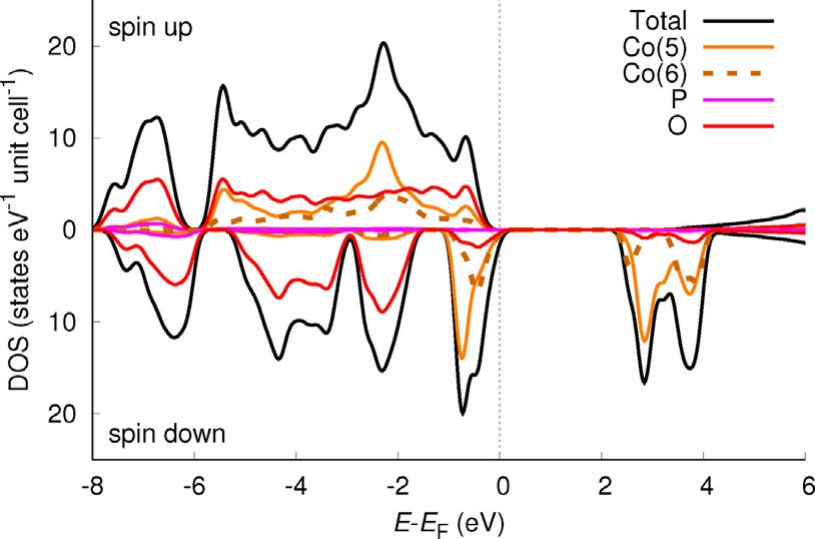
DFT‐calculated electronic density of states (DOS) and partial DOS (PDOS) for Co_3_(PO_4_)_2_. The Fermi level (*E*_F_) is set to 0 eV.

DFT calculations found the lowest energy when all Co ions were in high‐spin configurations. Figure 4 shows the calculated electronic density of states, DOS, for Co_3_(PO_4_)_2_. The high‐spin complexes cause the spin‐up (majority spin) and spin‐down (minority spin) components of the DOS to be quite different from each other. The spin‐down DOS features well‐localized Co d‐states near the valence‐band maximum (as seen from the partial DOS projected onto the individual Co ions, solid orange line and dashed brown line in Figure 4), with a one‐electron d–d‐gap calculated to be around 2.5 eV. The d–d transition energy is largely consistent with the experimentally measured absorption maximum for Co_3_(PO_4_)_2_ at 590 nm (2.10 eV);^24^ the HOMO–LUMO gap is here assumed to be only a first‐order approximation to the excitation energy. In contrast, the spin‐up valence band has significant contributions from both of the two inequivalent Co ions in the range from −6 to 0 eV (where the zero level is set to the Fermi energy at 0 K, *E*_F_).

The d‐orbital populations were estimated using the local coordinate systems around Co^5^ and Co^6^ from the multipole model. The DOS was projected onto the individual d‐orbitals and the populations were found by integrating the DOS up to the Fermi level. The resulting d‐orbital populations are given in Table 3 as percentage of the total d‐populations on the respective atoms. The table also gives the estimated d‐orbital populations from multipole refinement on the DFT‐calculated structure factors (MM‐DFT), as well as from the experimentally obtained structure factors (MM‐Expt). Table 3 shows overall satisfactory agreement between both populations, confirming that the high‐spin state for both Co ions found in the DFT calculations also corresponds to the experiment.

**Table 3:** d‐Orbital population as percentage of total d‐population on the respective atoms.

Atom	Orbital	MM‐Expt	MM‐DFT	DFT
Co^5^	d_*xz*_	23.4	23.3	25.2
	d_*yz*_	23.9	23.6	22.8
	d_*xy*_	17.8	17.7	19.0
	d${}_{x{}^{2}-y{}^{2}}$	19.1	19.2	17.2
	d${}_{z{}^{2}}$	15.7	16.2	15.7
Co^6^	d_*xz*_	22.2	20.6	19.9
	d_*yz*_	22.9	21.0	22.2
	d_*xy*_	22.3	25.5	25.3
	d${}_{x{}^{2}-y{}^{2}}$	16.5	16.3	16.2
	d${}_{z{}^{2}}$	16.1	16.7	16.4

The relative populations behave accordingly to simple crystal‐field theory: for the distorted trigonal‐bipyramidal complex around Co^[5by]^, the populations of d_*xz*_ and d_*yz*_ (≈24 % each) are larger than those of *d_xy_* and d${}_{x{}^{2}-y{}^{2}}$ (≈18 % each), which are larger than the population of d${}_{z{}^{2}}$ (≈16 %). For Co^6^, which has a distorted octahedral coordination environment, the populations of d_*xz*_, d_*yz*_, and d_*xy*_ (20–25 % from DFT calculations, ≈22 % from the experimental data) are greater than those of d${}_{x{}^{2}-y{}^{2}}$ and d${}_{z{}^{2}}$ (≈16 %). Although there is qualitative agreement in the d_*xz*_, d_*yz*_, and d_*xy*_ orbital populations between DFT, MM‐DFT, and MM‐Expt for Co^6^, we note that the populations obtained from the two DFT‐based methods have a noticeably higher population on d_*xy*_ (25 %) as compared to d_*xz*_ (20 %), whereas this is not the case from the experimental data (in which the orbitals have roughly the same fractional population of 22 %).

We explain this discrepancy as follows: given the 0, d_*yz*_ and d_*xy*_ orbitals at Co^6^ are occupied by only five electrons in a high‐spin state, there may be several nearly degenerate solutions to the Schrödinger equation and the present DFT calculations have converged to one of them. To obtain a truly accurate electronic description of the Co^6^ ion, it might therefore be necessary to make use of more advanced computational methods, such as multi‐reference methods. Still, even with this limitation for Co^6^, the agreement between the DFT calculations and the experiment is very good for Co^[5by]^.

### Maximally localized Wannier functions (MLWFs)

To visualize real‐space analogs of the electronic bands from the periodic DFT calculations, we calculated maximally localized Wannier functions centered on the Co atoms. The resulting MLWFs had clear connections to the d‐orbital populations, as well as the locations of the negative charge‐depletion regions and VSCCs around the Co ions. Given that all five spin‐up d‐orbitals are occupied, they form a roughly spherical ED. The two spin‐down orbitals add to the ED in particular directions, as can be seen from a comparison between the spin‐down MLWFs and the VSCCs.

**Figure 5:**
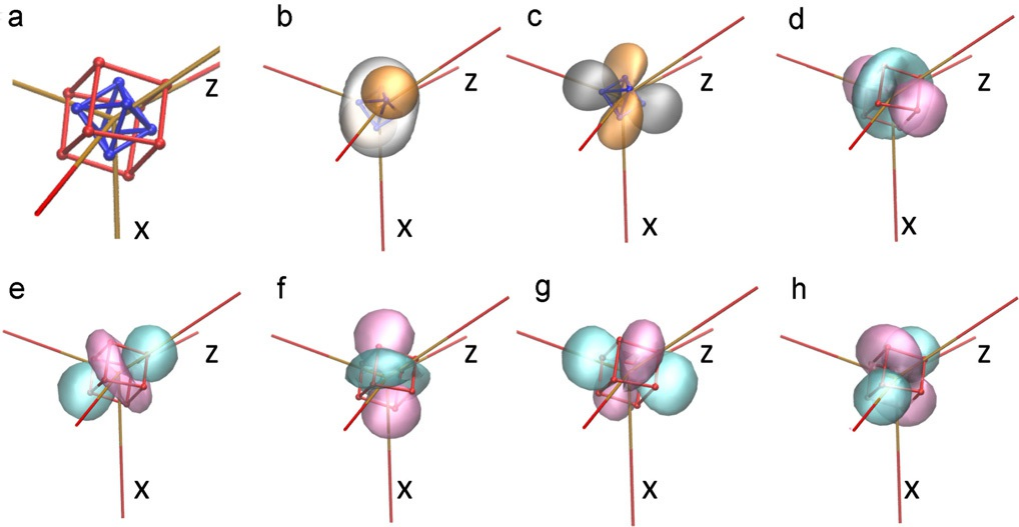
a) Positions of the VSCCs (blue) and negative charge depletion (red) around the fivefold coordinated Co^5^ ion as calculated from multipole refinements from DFT. b)–h) Maximally localized Wannier functions (isovalue ±0.2 Å^−3^) around Co^[5by]^. The chosen local coordinate system is indicated and is illustrated in more detail in Figure 3 a/b. b) and c) show spin‐down functions (orange and gray), together with the VSCCs (blue octahedron), d)–h) represent spin‐up functions (pink and cyan), together with charge depletion regions (red approximate cube).

Figure 5 a depicts the negative charge‐depletion points (red cube) at Co^[5by]^. Figure 5 b–c show the spin‐down MLWFs, pointing towards the directions of the VSCCs. Figure 5 d–h show the five spin‐up MLWFs centered on Co^[5by]^. Interestingly, there is a clear connection between the charge‐depletion points and the orientation of the spin‐up MLWFs. In particular, the two MLWFs in Figure 5 d–g, which have shapes that are reminiscent of atomic d${}_{z{}^{2}}$ orbitals, have one of their lobes pointing towards the charge‐depletion point along one of the equatorial Co−O bonds, and the other lobe pointing towards one of the charge‐depletion points that does not lie along any Co−O bond. In fact, this latter type of charge depletion points towards a small void in the crystal. The MLWFs for Co^6^ are provided in the Supporting Information.

**Figure 6:**
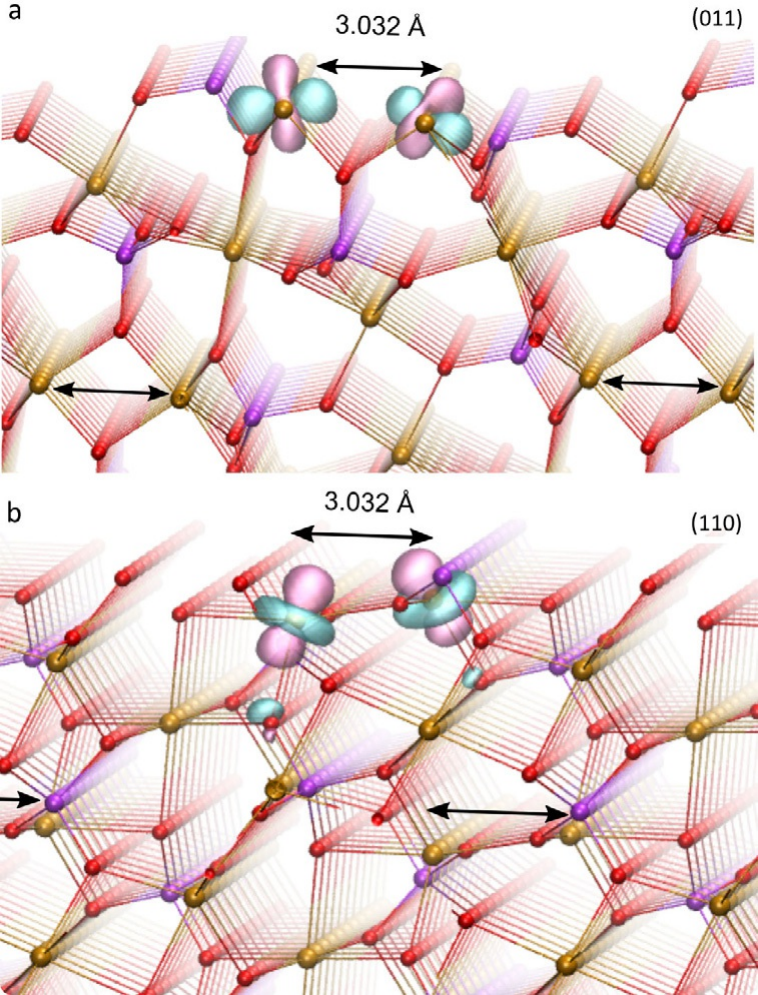
Side views of (a) the (011) face, and (b) the (110) face, of Co_3_(PO_4_)_2_ shown as ball‐and‐stick models. Colors: Co is brown, O is red, and P is magenta. The arrows indicate Co atom pairs with a short distance 3.032 Å between them. The iso‐surfaces show two of the MLWFs (±0.08 Å^−3^) from Figure 5 f for the (011) face and Figure 5 d for the (110) face that have their upward‐pointing lobes pointing towards negative charge‐depletion regions around the respective Co ions. Charge analysis was carried out for the bulk crystal.

We also consider the charge‐depletion regions and MLWFs around two edge‐sharing Co^5^ ions, and correlate these regions to the different orientation at various Co_3_(PO_4_)_2_ faces. The two (011) and (110) faces of cobalt orthophosphate expose nearby Co^5^ ions. Figure 6 a shows a side view of the Co_3_(PO_4_)_2_ (011) face. The arrows indicate the two edge‐sharing Co^5^ atoms in the bulk; at the (011) face (top layer), the two ions only share one ligand in the sub‐face layer. The MLWF from Figure 5 is shown superimposed on two of the face Co^5^ ions. The blue lobes pointing out of the face are pointing along negative charge‐depletion regions. Similarly, at the (110) face (Figure 6 b), the MLWF from Figure 5 d have lobes pointing out of the face, towards negative charge‐depletion regions. Considering cooperativity between two nearby Co ions as for example, stabilizing low oxidation states^25^ or molecular conductors of electricity,^26^ this pictures would suggest the crystal plane (011) as clearly catalytically more active than any other.

### Considering catalytic abilities from ED

Phosphates, which contain cobalt and other transition metals, are known to catalyze organic reactions and are therefore in the spotlight of current research. The oxidation of styrene, for example, was realized using a nickel–cobalt catalyst.^27^ The reduction of *p*‐nitrophenol to *p*‐aminophenol can be effectively catalyzed by nanosized solid solutions of copper/cobalt molybdate and chromium phosphate.^28^ For a detailed study of the cobalt‐substituted calcium phosphate catalyst, which catalyzes oxidative dehydration and dehydrogenation, Legrouri et al.^29^ used propane‐2‐ol as a benchmark substrate for characterizing acid and basic properties of solid‐state catalysts (Scheme 1, see above). The maximum catalytic effect was found at a cobalt content of 0.32, which also represents the maximum solubility of Co in calcium phosphate. The reactivity for ethane, propane, butan‐2‐ol, and propanol was studied by Aaddane et al.^5^ and plausibility for the reactivity was provided.

Considering the reaction of butan‐2‐ol with oxygen to butene and butenone, at least one of the two reactants must be chemisorbed at the catalyst's surface and thus be activated. If the butanol is adsorbed over several atoms on the surface due to its size, the negative oxygen atom most likely coordinates through the two oxygen lone pairs to an electronically depleted site of cobalt atoms and the positively polarized methyl hydrogen atoms to a nearby basic phosphate site. If we assume differences in the heterogeneous catalytic ability of the solid‐state faces in cobalt phosphate we need to establish criteria to identify effective catalytic sites.^30^ Given that the crystal structure of cobalt phosphate shows two differently coordinated metal sites, the first question to be asked is whether both Co atoms are equally active, and if not, what causes the differences. If the plain coordination number of the cobalt atoms is considered, the saturated Co^6^ most likely is not tremendously active as it lacks a vacant site for the substrate to interact with. Figures 3 g and 3 h clearly show that there are no free sites at Co^6^, whereas Co^5^ holds three of them. However, it has to be kept in mind that Co‐atoms exposed at the surface inevitably render free coordination sites. Depending on how exposed they are on the surface, the number of free coordination sites also varies, which affects the probability of chemisorption. However, statistically speaking, Co^5^ would still have to have more free coordination sites than Co^6^ at the surface, because they already have three more vacant sites in the bulk. In addition, the present distortion of the geometry will promote catalysis.^31^

**Figure 7:**
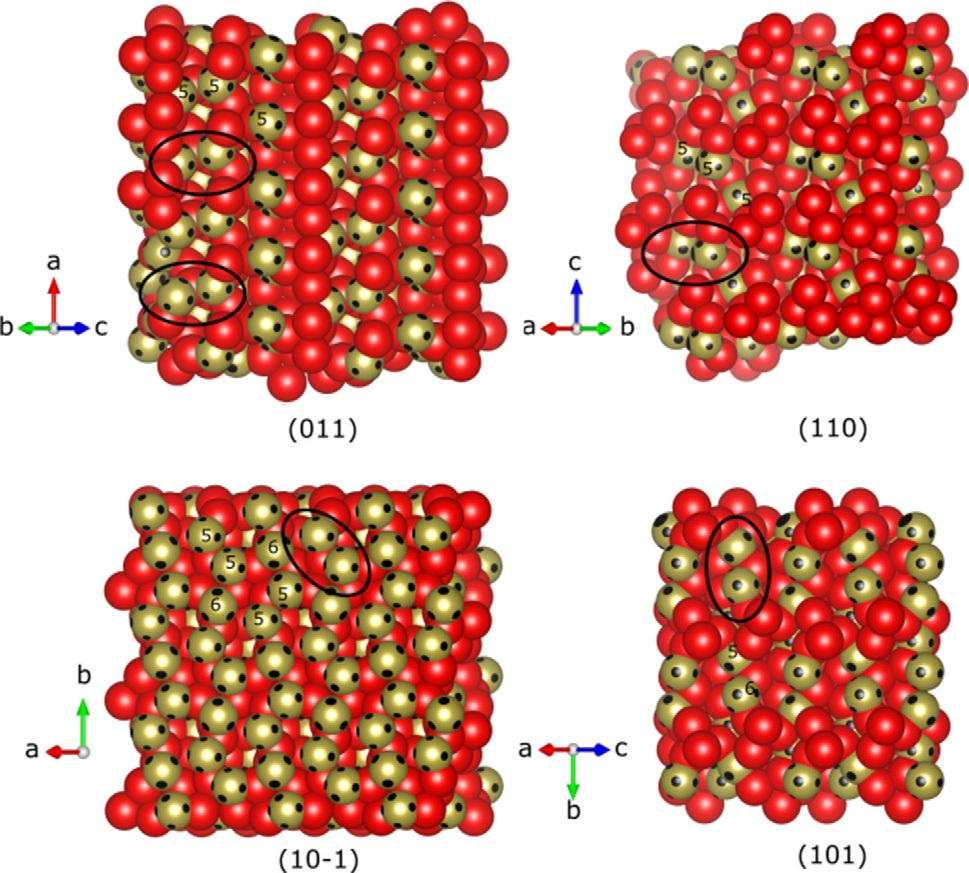
Space‐filled view of the four most stable crystal planes of cobalt phosphate according to the Bravais, Friedel, Donnay, and Harker theory. Red: oxygen, bronze: cobalt, phosphorus is hidden inside the O_4_ tetrahedra.

When for example, C−C bonds are activated in a substrate by rhodium and nickel catalysts, it is proven that this is a structurally sensitive reaction that takes place on coordinatively unsaturated atom pairs of the d‐metals on corners or edges of minute crystallites.^2^ Thinking in terms of concepts of homometallic cooperativity,^32^ short distances between the metal centers are needed. In the crystal structure of cobalt phosphate, there are such short distances between two fivefold coordinated edge‐sharing Co‐ions (3.032 Å) (Figure 2 b) and between a fivefold and a sixfold Co‐ion (3.145 Å). According to the Bravais, Friedel, Donnay, and Harker theory,^33^ Figure 7 shows the four most likely low‐index crystal faces with Co⋅⋅⋅Co cooperative sites. The layer is chosen such that no covalent bonds are broken in the phosphate and cobalt atoms appear on the surface. The positions of electronically depleted areas found from the multipole refined Laplacians are superimposed onto the surface of the cobalt atoms as black areas. At the (011) face only Co^5^ and O atoms are visible, where the Co atoms occur isolated or in pairs with a short interatomic distance of 3.032 Å. The Co^5^ atoms face each other in pairs, each exposing three and four vacant coordination sites. The pairs at the edges even have up to six vacant coordination sites per Co atom. They are perfectly arranged to serve as adsorption sites for the two oxygen lone pairs of the butanol molecules, which then σ‐bridges two Co atoms.

Looking at the other lattice planes from a similar point of view, plane (110) shows similar properties to (011), but all Co‐atoms are slightly deeper embedded in the surface thus they are much more difficult to be reached by the butanol oxygen atom. At the (10‐1) face exclusively five‐ and sixfold coordinated cobalt atoms are exposed. Due to the solemnly electronically depleted sites at this surface, the chemisorption of a molecule with both, electron‐rich and electron‐poor regions like butan‐2‐ol is much more difficult (Figure 7).The slightly positive σ‐hydrogen atom H^3^ (Scheme 1) would probably not find a more basic nearby phosphate site to be activated. The (101) plane also displays Co‐atom pairs with short metal–metal distances but they occur between five‐ and sixfold coordinated Co atom. Furthermore, they are not as well arranged as at the (011) plane. Hence, we conclude that (011) plane is the catalytically most active.

Finally, we note that the above arguments were made based on the ED for the bulk crystal. To verify that the predicted adsorption of for example butan‐2‐ol onto the Co^5^ at the (011) surface is favorable, we used DFT to calculate the adsorption energy of butan‐2‐ol onto several different adsorption sites for two different surface terminations of the (011) surface and found the predicted adsorption site to be indeed the most favorable (see the Supporting Information) as expected, confirming that the bulk ED can be used as a tool to qualitatively predict adsorption behavior at the surface. Nevertheless, the surface structure of a real catalyst may deviate from bulk‐truncated cuts, and so based on this analysis we cannot fully exclude the presence of additional relevant active sites and surface facets. However, insofar as the catalyst surface structure resembles any of the low‐index surfaces, the (011) surface has the right structural features and ED for catalytic conversion.

## Conclusions

An extensive characterization of cobalt phosphate using topological analysis and theoretical methods for experimental and theoretical electron‐density distribution was performed. The P−O bonds were identified as polar single bonds in which the BCP is considerably shifted to the phosphorus atom. The Co−O bonds can be described as closed‐shell interaction. The calculated MLWFs have a clear connection to the d‐orbital occupation, as well as the position of the charge‐depletion centers. In the solid state, the two independent cobalt atoms exhibit different numbers of charge depletion sites in the valence shell. Co^5^ has, in addition to the charge depletions directed towards the negatively charged phosphate oxygen atoms, three additional vacant coordination sites. This suggests that the nucleophilic attack on Co^5^ is much more likely. These depletions, congruent with the unoccupied or partially occupied d‐orbitals, were analyzed on the face of different solid‐state surfaces. It has been shown that the (011) face is the most suitable for catalytic oxidative dehydrogenation or dehydrogenation, showing Co^[5by]^⋅⋅⋅Co^5^ cooperativity. There are sufficient acidic and basic sites for the chemisorption of butan‐2‐ol available. On this face Co^5^ ions and phosphate ions are the most accessible; therefore, this surface is best equipped to activate both, the C−H and O−H bonds. Furthermore, the lobes of the MLWFs of both cooperative Co^5^ atoms face each other to address both oxygen lone pairs of the substrate. The investigations show that the concerted but independent experimental and theoretical determination of charge‐density distribution is a valuable tool to rationalize catalytic active materials.

## Experimental Section

**High‐resolution structure determination**: Cobalt orthophosphate was purchased as powder from Alfa Aesar. Given that it is not soluble in any conventional organic solvents, it was crystallized from the melt. The solid was heated to 1180 °C under air atmosphere in a chamber oven, then cooled to 500 °C, tempered for one hour and finally cooled down to room temperature. The crystallization approach known from the Ref. 7 in slightly modified form was also tested, but did not produce better single crystals. A violet single crystal of 45×90×100 μm in size was mounted at room temperature on the goniometer using inert perfluorinated polyether oil and cooled to 100 K.

High‐resolution X‐ray diffraction data were collected on a Bruker SMART APEX II diffractometer based on D8 three‐circle goniometer system using an Incoatec microfocus AgKα source (IμS) and Incoatec QUAZAR mirror optics.^34^ A Bruker APEX II CCD detector was used to record the diffracted intensities at *λ*=0.56086 Å. Data reduction was carried out with SAINT^35^ (version 8.30C) from the APEX2^36^ program package in which the integration box sizes were refined for every run using a standard procedure. The data were scaled and equivalent reflections were merged with SADABS^37^ (version 2016/2). An empirical absorption correction for a strong absorber was also performed with SADABS. Afterwards the structure was solved with SHELXT^38^ using the direct methods and refined by full‐matrix least square against *F* ^2^ using SHELXL^39^ (version 2018/1) by means of the graphical user interface SHELXle.^40^ Topological analysis according to the Quantum Theory of Atoms in Molecules^12^ (QTAIM) was performed using the XDPROP and TOPXD programs included in the XD package.^41^ Crystallographic details are provided in Table S1‐1, Supporting Information.

**DFT calculations**: Periodic DFT calculations were performed on the experimentally obtained Co_3_(PO_4_)_2_ crystal structure using the WIEN2k program package version 17.1.^42^ The composition of the conventional unit cell is Co_6_(PO_4_)_4_. Spin‐polarized calculations were performed using the PBE+U functional (*U_eff_*=*U*−*J=*3 eV). The value of *U_eff_* was chosen on the basis of previous studies for cobalt oxides and hydroxides.^43^ Although the value of *U_eff_* affects the calculated (one‐electron) band gap and dd transition level, we have verified that other choices (*U_eff_*=1 eV, *U_eff_*=4 eV) do not affect the qualitative results of this study, in particular as pertains to the shape and orientation of the maximally localized Wannier functions^44^ (MLWFs). The calculations were performed using the linearized augmented plane wave (LAPW) method with *RK*_max_=8.5. The Brillouin zone was sampled with a 19×11×13 k‐point grid, corresponding to 744 irreducible k‐points; the total energy was then converged within 3 meV as compared to a 9×5×6 k‐point grid. The lowest‐energy structure was found when all Co ions were in high‐spin configurations. We do not consider any magnetic ordering or relative alignment of spins, because the experiments in the current study were conducted at *T*=100 K at which temperature the compound is paramagnetic. MLWFs were calculated using the WIEN2k Wannier interface to the wannier90 program^45^ (i) by projecting the 12 highest occupied spin‐down bands (two occupied bands per Co ion) onto the Co d‐orbitals, and (ii) by projecting the highest 62 occupied spin‐up bands onto the 30 Co d‐orbitals in the conventional unit cell. We attempted several different rotations for the initial projections, but found that all of them converged to the same final result. The MLWFs were plotted using VMD.^46^ The X‐ray structure factors used as input for the multipole model were calculated for reflections up to sin *θ* *λ*<1.61 Å^−1^ using the WIEN2k lapw3 program. We also performed periodic surface DFT calculations using Quantum ESPRESSO,^47^ and molecular DFT calculations using ADF.^48^ The details of those calculations are given in the Supporting Information.

## Conflict of interest

The authors declare no conflict of interest.

## Supporting information

As a service to our authors and readers, this journal provides supporting information supplied by the authors. Such materials are peer reviewed and may be re‐organized for online delivery, but are not copy‐edited or typeset. Technical support issues arising from supporting information (other than missing files) should be addressed to the authors.

Supplementary

## References

[1a] B. Cornils , W. A. Herrmann , Applied Homogenous Catalysis with Organometallic Compounds, VCH, Weinheim, 1996;

[1b] P. Gandeepan , T. Müller , D. Zell , G. Cera , S. Warratz , L. Ackermann , Chem. Rev. 2019, 119, 2192; 30480438 10.1021/acs.chemrev.8b00507

[1c] S. Santoro , S. I. Kozhushkov , L. Ackermann , L. Vaccaro , Green Chem. 2016, 18, 3471.

[2] W. Reschetilowski , Einführung in die Heterogene Katalyse, Springer Spektrum, Berlin, 2015.

[3a] C. Gatti , P. Macchi , Modern Charge Density Analysis, Springer, Heidelberg, London, New York, 2012;

[3b] U. Flierler , D. Stalke , Struct. Bonding (Berlin) 2012, 146, 1;

[3c] D. Stalke , Struct. Bonding (Berlin) 2016, 169, 57;

[3d] P. Coppens , Angew. Chem. Int. Ed. 2005, 44, 6810; 10.1002/anie.200501734 16187393

[3e] C. Gatti , Z. Kristallogr. 2005, 220, 399;

[3f] A. Genoni , L. Bučinský , N. Claiser , J. Contreras-García , B. Dittrich , P. M. Dominiak , E. Espinosa , C. Gatti , P. Giannozzi , J.-M. Gillet , D. Jayatilaka , P. Macchi , A. Ø. Madsen , L. Massa , C. F. Matta , K. M. Merz Jr. , P. N. H. Nakashima , H. Ott , U. Ryde , K. Schwarz , M. Sierka , S. Grabowsky , Chem. Eur. J. 2018, 24, 10881; 29488652 10.1002/chem.201705952

[3g] D. Stalke , Chem. Eur. J. 2011, 17, 9264. 21717511 10.1002/chem.201100615

[4] L. Pauling , J. Am. Chem. Soc. 1932, 54, 988.

[5] A. Aaddane , M. Kacimi , M. Ziyad , Catal. Lett. 2001, 73, 47.

[6] A. G. Nord , D. P. Novak , A. Borgan , T. Østvold , A. Bjørseth , D. L. Powell , Acta Chem. Scand. A 1974, 28, 150.

[7] J. B. Anderson , E. Kostiner , M. C. Miller , J. R. Rea , J. Solid State Chem. 1975, 14, 372.

[8] G. Berthet , J. C. Joubert , E. F. Bertaut , Z. Kristallogr. 1972, 136, 98.

[9] A. W. Addison , T. N. Rao , J. Reedijk , J. van Rijn , G. C. Verschoor , J. Chem. Soc. Dalton Trans. 1984, 1349.

[10a] F. Machatschki , Monatsh. Chem. 1947, 77, 333;

[10b] J. Lima-de-Faria , E. Hellner , F. Liebau , E. Makovicky , E. Parthé , Acta Crystallogr. Sect. A 1990, 46, 1.

[11] N. K. Hansen , P. Coppens , Acta Crystallogr. Sect. B 1978, 34, 909.

[12] R. F. W. Bader , Atoms in Molecules. A Quantum Theory, Clarendon Press, Oxford, New York, 1990.

[13] W. Jones , March , Norman , Henry , Theoretical Solid State Physics. Volume 1: Perfect Lattices in Equilibrium, Dover Publications Inc., New York, 1985.

[14] J. A. Dobado , H. Martínez-García , Molina , M. R. Sundberg , J. Am. Chem. Soc. 1998, 120, 8461.

[15] D. Leusser , J. Henn , N. Kocher , B. Engels , D. Stalke , J. Am. Chem. Soc. 2004, 126, 1781. 14871110 10.1021/ja038941+

[16a] M. C. Durrant , Chem. Sci. 2015, 6, 6614; 29999045 10.1039/c5sc04866d PMC6007126

[16b] R. D. Harcourt , T. M. Klapötke , Chem. Sci. 2016, 7, 3443;29999045

[16c] M. C. Durrant , Chem. Sci. 2016, 7, 3448. 29999044 10.1039/c6sc00859c PMC6007093

[17] M. S. Schmøkel , S. Cenedese , J. Overgaard , M. R. V. Jørgensen , Y.-S. Chen , C. Gatti , D. Stalke , B. B. Iversen , Inorg. Chem. 2012, 51, 8607. 22834961 10.1021/ic301372m

[18] M. Fugel , L. A. Malaspina , R. Pal , S. P. Thomas , M. W. Shi , M. A. Spackman , K. Sugimoto , S. Grabowsky , Chem. Eur. J. 2019, 25, 6523. 30759315 10.1002/chem.201806247

[19] D. Cremer , E. Kraka , Croat. Chem. Acta 1984, 56, 1259.

[20] E. Espinosa , I. Alkorta , J. Elguero , E. Molins , J. Chem. Phys. 2002, 117, 5529.

[21] R. J. Gillespie , I. Hargittai , The VSEPR model of molecular geometry, Allyn and Bacon, Boston, London, 1991.

[22] L. Krause , B. Niepötter , C. J. Schürmann , D. Stalke , R. Herbst-Irmer , IUCrJ 2017, 4, 420. 10.1107/S2052252517005103 PMC5571805 28875029

[23a] G. V. Gibbs , M. A. Spackman , D. Jayatilaka , K. M. Rosso , D. F. Cox , J. Phys. Chem. A 2006, 110, 12259; 17078623 10.1021/jp062992m

[23b] G. V. Gibbs , R. T. Downs , D. F. Cox , K. M. Rosso , N. L. Ross , A. Kirfel , T. Lippmann , W. Morgenroth , T. D. Crawford , J. Phys. Chem. A 2008, 112, 8811. 18714960 10.1021/jp804280j

[24] A. Schmidt , Dissertation, Justus-Liebig-Universität Gießen, Gießen, 2002.

[25] K. C. Mondal , P. P. Samuel , H. W. Roesky , E. Carl , R. Herbst-Irmer , D. Stalke , B. Schwederski , W. Kaim , L. Ungur , L. F. Chibotaru , M. Hermann , G. Frenking , J. Am. Chem. Soc. 2014, 136, 1770. 24437683 10.1021/ja4123285

[26] S. R. Madsen , M. K. Thomsen , S. Scheins , Y.-S. Chen , N. Finkelmeier , D. Stalke , J. Overgaard , B. B. Iversen , Dalton Trans. 2014, 43, 1313. 24192864 10.1039/c3dt52035h

[27] D. Gao , Q. Gao , Microporous Mesoporous Mater. 2005, 85, 365.

[28] T. K. Ghorai , D. Dhak , A. Azizan , P. Pramanik , Mater. Sci. Eng. B 2005, 121, 216.

[29a] A. Legrouri , J. Lenzi , M. Lenzi , React. Kinet. Catal. Lett. 1997, 62, 313;

[29b] A. Legrouri , J. Lenzi , M. Lenzi , React. Kinet. Catal. Lett. 1998, 65, 227.

[30] H. S. Taylor , Proc. R. Soc. London Ser. A 1925, 108, 105.

[31] Y. Sohtome , G. Nakamura , A. Muranaka , D. Hashizume , S. Lectard , T. Tsuchimoto , M. Uchiyama , M. Sodeoka , Nat. Commun. 2017, 8, 14875. 28383035 10.1038/ncomms14875 PMC5384211

[32] J. A. Mata , F. E. Hahn , E. Peris , Chem. Sci. 2014, 5, 1723.

[33] J. D. H. Donnay , D. Harker , Am. Mineral. 1937, 22, 446.

[34] T. Schulz , K. Meindl , D. Leusser , D. Stern , J. Graf , C. Michaelsen , M. Ruf , G. M. Sheldrick , D. Stalke , J. Appl. Crystallogr. 2009, 42, 885.

[35] Bruker SAINT v8.30C. Bruker AXS Inst. Inc., WI, USA, Madison, **2013**.

[36] Bruker APEX v2011.4-1. Bruker AXS Inst. Inc., WI, USA, Madison, **2011**.

[37] L. Krause , R. Herbst-Irmer , D. Stalke , J. Appl. Crystallogr. 2015, 48, 1907. 10.1107/S1600576714022985 PMC4453166 26089746

[38] G. M. Sheldrick , Acta Crystallogr. Sect. A 2015, 71, 3.

[39] G. M. Sheldrick , Acta Crystallogr. Sect. C 2015, 71, 3. 10.1107/S2053273314026370 PMC4283466 25537383

[40] C. B. Hübschle , G. M. Sheldrick , B. Dittrich , J. Appl. Crystallogr. 2011, 44, 1281. 22477785 10.1107/S0021889811043202 PMC3246833

[41] A. V. Volkov, P. Macchi, L. J. Farrugia, C. Gatti, P. Mallinson, T. Richter, T. Koritsanszky, *XD2006*, **2006**.

[42] P. Blaha , K. Schwarz , G. Madsen , D. Kvasnicka , J. Luitz , WIEN2k. An Augmented Plane Wave Plus Local Orbitals Program for Calculating Crystal Properties, Technische Universität Wien, 2001.

[43a] L. Wang , T. Maxisch , G. Ceder , Phys. Rev. B 2006, 73, 603; 10.1103/PhysRevLett.97.155704 17155339

[43b] C. Rödl , F. Fuchs , J. Furthmüller , F. Bechstedt , Phys. Rev. B 2009, 79, 1996;

[43c] J. Chen , A. Selloni , J. Phys. Chem. C 2013, 117, 20002;

[43d] B. S. Youmbi , F. Calvayrac , Surf. Sci. 2014, 621, 1.

[44] N. Marzari , D. Vanderbilt , Phys. Rev. B 1997, 56, 12847.

[45] A. A. Mostofi , J. R. Yates , G. Pizzi , Y.-S. Lee , I. Souza , D. Vanderbilt , N. Marzari , Comput. Phys. Commun. 2014, 185, 2309–2310.

[46a] J. Stone, *M.Sc. thesis*, University of Missouri-Rolla **1998**;

[46b] W. Humphrey , A. Dalke , K. Schulten , J. Mol. Graph. 1996, 14, 33–8, 27–8. 8744570 10.1016/0263-7855(96)00018-5

[47a] P. Giannozzi , O. Andreussi , T. Brumme , O. Bunau , M. Buongiorno Nardelli , M. Calandra , R. Car , C. Cavazzoni , D. Ceresoli , M. Cococcioni , N. Colonna , I. Carnimeo , A. Dal Corso , S. de Gironcoli , P. Delugas , R. A. DiStasio Jr. , A. Ferretti , A. Floris , G. Fratesi , G. Fugallo , R. Gebauer , U. Gerstmann , F. Giustino , T. Gorni , J. Jia , M. Kawamura , H.-Y. Ko , A. Kokalj , E. Küçükbenli , M. Lazzeri , M. Marsili , N. Marzari , F. Mauri , N. L. Nguyen , H.-V. Nguyen , A. Otero-de-la-Roza , L. Paulatto , S. Poncé , D. Rocca , R. Sabatini , B. Santra , M. Schlipf , A. P. Seitsonen , A. Smogunov , I. Timrov , T. Thonhauser , P. Umari , N. Vast , X. Wu , S. Baroni , J. Phys. Condens. Matter 2017, 29, 465901; 29064822 10.1088/1361-648X/aa8f79

[47b] P. Giannozzi , S. Baroni , N. Bonini , M. Calandra , R. Car , C. Cavazzoni , D. Ceresoli , G. L Chiarotti , M. Cococcioni , I. Dabo , A. Dal Corso , S. de Gironcoli , S. Fabris , G. Fratesi , R. Gebauer , U. Gerstmann , C. Gougoussis , A. Kokalj , M. Lazzeri , L. Martin-Samos , N. Marzari , F. Mauri , R. Mazzarello , S. Paolini , A. Pasquarello , L. Paulatto , C. Sbraccia , S. Scandolo , G. Sclauzero , A. P Seitsonen , A. Smogunov , P. Umari , R. M Wentzcovitch , J. Phys. Condens. Matter 2009, 21, 395502. 21832390 10.1088/0953-8984/21/39/395502

[48a] Baerends, E. J. et al., ADF2017, SCM, Theoretical Chemistry, Vrije Universiteit, Amsterdam;

[48b] C. Fonseca Guerra , J. G. Snijders , G. te Velde , E. J. Baerends , Theor. Chem. Acc. 1998, 99, 391;

[48c] G. te Velde , F. M. Bickelhaupt , E. J. Baerends , C. Fonseca Guerra , S. J. A. van Gisbergen , J. G. Snijders , T. Ziegler , J. Comput. Chem. 2001, 22, 931.

