# Supplementary material for: New Insights into the Catalytic Activity of Cobalt Orthophosphate Co3(PO4)2 from Charge Density Analysis
Source: Chemistry. 2019 Nov 8;25(69):15786–94. doi: 10.1002/chem.201902303 (PMC6916324; doi:10.1002/chem.201902303)
Supplement: Supplementary file 1 — Supplementary [file CHEM-25-15786-s001.pdf]

CHEMISTRY
A European Journal

Supporting Information

New Insights into the Catalytic Activity of Cobalt Orthophosphate $Co_3(PO_4)_2$ from Charge Density Analysis

Helena Keil,[a] Matti Hellström,[b, c] Claudia Stückl,[a] Regine Herbst-Irmer,[a] Jörg Behler,[b] and Dietmar Stalke*[a]

chem_201902303_sm_miscellaneous_information.pdf

Content

S1. Crystallographic details 2
S2. Multipole Refinement Strategy 2
S3. Data quality for Multipole Refinement 5
S3.1. Experimental data 5
S3.2. DFT data 6
S4. Properties 7
S5. Plots for comparison of MM-Expt with MM-DFT Refinement. 9
S6. Maximally localized Wannier functions 10
S7. Non-periodic DFT calculations 11
S8. Site views of the crystal planes 13
S9. Plane-wave bulk and surface DFT calculations 13

S1. Crystallographic details

Table S1-1 Crystallographic details at 100 K.

Formula	$Co_3(PO_4)_2$	**IAM Refinement**	
λ (Å)	0.56086 (Ag)	Data / restraints / parameters	11215 / 0 / 62
Crystal system, space group	Monoclinic, $P2_1/n$	Final R indices [I>2σ(I)]	R1 = 0.0143,
a (Å)	5.068(2)		wR2 = 0.0364
b (Å)	8.340(3)	R indices (all data)	R1 = 0.0165,
c (Å)	7.554(2)		wR2 = 0.0372
β (°)	94.13(2)	Extinction coefficient	0.0170(13)
Crystal size (µm)	~45 x 90 x 100	$\Delta\rho_{max}$, $\Delta\rho_{min}$ (e Å^{-3})	2.240, -1.767
Volume (Å^3)	318.46(19)	**Multipole Refinement Experimental Data**	
Density (Mg/m^3)	3.825	Data / parameters	10976 / 128
µ (mm^{-1})	4.231	$R(F^2)$, $Rw(F^2)$	0.0121, 0.0126
F(000)	350	GOF	1.138
(sin ϑ/λ)min, (sin ϑ/λ)max (Å^{-1})	0.089, 1.614	Extinction coefficient	0.000737(11)
Ref. meas., Ref. unique	306674, 11218	$\Delta\rho_{max}$, $\Delta\rho_{min}$ (e Å^{-3})	0.658, -0.518
R_{int}	0.0315	**Multipole Refinement Theoretical Data**	
		Data / parameters	10838 / 67
		$R(F^2)$, $Rw(F^2)$	0.0041, 0.0028
		GOF	4.048
		$\Delta\rho_{max}$, $\Delta\rho_{min}$ (e Å^{-3})	0.559, -0.581

Table S1-2. Bonds and angles with their standard deviations for both Co atoms (100 K). Oxygen atoms marked with an X are generated by the symmetry code. X1: -1+x, +y, +z; X2: -1/2+x, 1/2-y, -1/2+z; X3: -x, 1-y, 1-z; X4: 1-x, 1-y, 1-z; X5: 1/2-x, 1/2+y, 3/2-z; X6: 3/2-x, -1/2+y, 3/2-z; X7: 1/2+x, 1/2-y, -1/2+z; X8: 1+x, +y, +z.

$Co^{[5by]}$ - O	*d* [Å]	$Co^{[6o]}$ - O	*d* [Å]
O1	1.97728(12)	O1_X1	2.14380(12)
O2_X8	2.22177(13)	O2	2.05631(12)
O3_X8	2.01884(12)	O3_X5	2.16934(12)
O4_X6	1.98881(12)		
O4_X4	2.00786(12)		

O - Co - O			∠ [°]
O1	$Co^{[5by]}$	O2_X8	81.023(5)
O1	$Co^{[5by]}$	O3_X8	127.034(5)
O1	$Co^{[5by]}$	O4_X6	110.880(5)
O1	$Co^{[5by]}$	O4_X7	100.440(5)
O2_X8	$Co^{[5by]}$	O3_X8	70.082(5)
O2_X8	$Co^{[5by]}$	O4_X6	167.309(5)
O2_X8	$Co^{[5by]}$	O4_X7	92.407(5)
O3_X8	$Co^{[5by]}$	O4_X6	104.164(5)
O3_X8	$Co^{[5by]}$	O4_X7	123.499(5)
O4_X6	$Co^{[5by]}$	O4_X7	81.335(5)
O1_X1	$Co^{[6o]}$	O2	81.196(5)
O1_X1	$Co^{[6o]}$	O3_X5	93.583(5)
O1_X1	$Co^{[6o]}$	O3_X2	86.417(5)
O1_X4	$Co^{[6o]}$	O2	98.804(5)
O2	$Co^{[6o]}$	O3_X5	95.150(5)
O2	$Co^{[6o]}$	O3_X2	84.850(5)

S2. Multipole Refinement Strategy

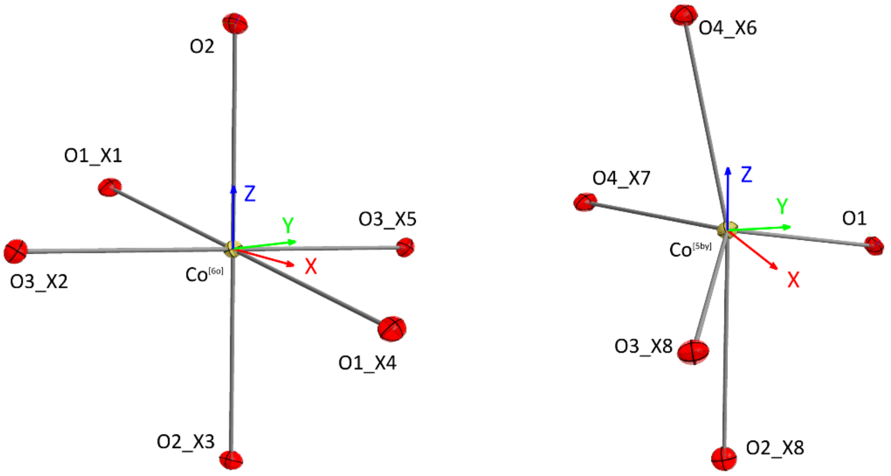

Figure S2-1. Local coordinate systems for Co[6o] and Co[5by].

Table S2-1. Local coordinate system

ATOM	ATOM0	AX1	ATOM1	ATOM2	AX2	KAP	START SITESYM	END SITESYM	CHEMCON
Co(6)	O2	Z	Co1	DUM0	Y	1	234ZmYmX (cyl)	noSym	
Co(5)	O2	Z	Co2	DUM5	X	1	24ZmYmX (4mm)	noSym	
P1	O2	Z	P1	O1	Y	2	3ZmX	3ZmX	
O1	P1	Z	O1	Co2	Y	3	234ZmYmX (cyl)	mX	
O2	P1	Z	O2	DUM1	Y	3	234ZmYmX (cyl)	mX	O1
O3	P1	Z	O3	DUM3	Y	3	234ZmYmX (cyl)	mX	O1
O4	P1	Z	O4	DUM2	Y	3	234ZmYmX (cyl)	mX	O1

Table S2-2. Fractional coordinates for dummy atoms

DUM	x	y	z
0	0.051990	0.637572	0.743521
1	0.095398	0.140940	0.613902
2	0.404602	0.640940	0.886098
3	0	0.5	1.0
5	1.195400	0.140900	0.713900

Table S2-3. Strategy for Experimental data. The final strategy goes up to and including step 11. The last two steps have been omitted as they lead to over-fitting. See Figure S1-1. Abbreviations: **Sf**: scale factor; **MP**: multipoles; **M**: monopoles; **D**: dipoles; **Q**: quadrupoles; **O**: octupoles; **H**: hexadecapoles, **U2**: Uij, **k**: kappa, **k'**: kappa prime **noSym**: no local symmetry constraints, **GOF**. Goodness of Fit; **low-res data**: data < 0.5 sin(th)/l. Every new added parameter is marked in red.

Step	Refined Parameter	Data	MP-Param.	Ratio low-res Data to MP, k & k'	Param.	Ratio Data to Param.	GOF	R(F^2)
1	Sf	10976	0	0	1	10976	3.623	0.0323
2	Sf, Exti	10976	0	0	2	5488	3.173	0.0246
3	Sf, Exti, DQOH	10976	16	21.1	18	609.8	2.476	0.0218
4	Sf, Exti, DQOH, U	10976	16	21.1	60	182.9	2	0.0165
5	Sf, Exti, MDQOH, U	10976	22	15.3	65	168.9	1.458	0.0149
6	Sf, Exti, MDQOH, U, XYZ	10976	22	15.3	83	132.2	1.415	0.0146
7	Sf, Exti, MDQOH, U, XYZ, k	10976	22	13.5	86	127.6	1.343	0.0141
8	Sf, Exti, MDQOH, U, XYZ, k, 4mm -> noSym [Co(5)]	10976	43	7.3	107	102.6	1.179	0.0125
9	Sf, Exti, MDQOH, U, XYZ, k, nosym [Co(5)], cyl -> noSym [Co(6)]	10976	54	5.9	118	93	1.157	0.0122
10	Sf, k'	10976	0	84	4	2744	1.146	0.0122
11	Sf, Exti, MDQOH, U, XYZ, k, nosym [Co(6), Co(5)],	10976	64	5	128	85.8	1.138	0.0121

	cyl -> mX [O]							
12	Sf, Exti, MDQOH, U, XYZ, k, nosym [Co(6), Co(5)], mX -> noSym [O]	10976	74	4.4	138	79.5	1.133	0.0121
13	Sf, Exti, MDQOH, U, XYZ, k, nosym [Co(6), Co(5), O], noCon [O]	10976	149	2.2	213	51.5	1.096	0.0118

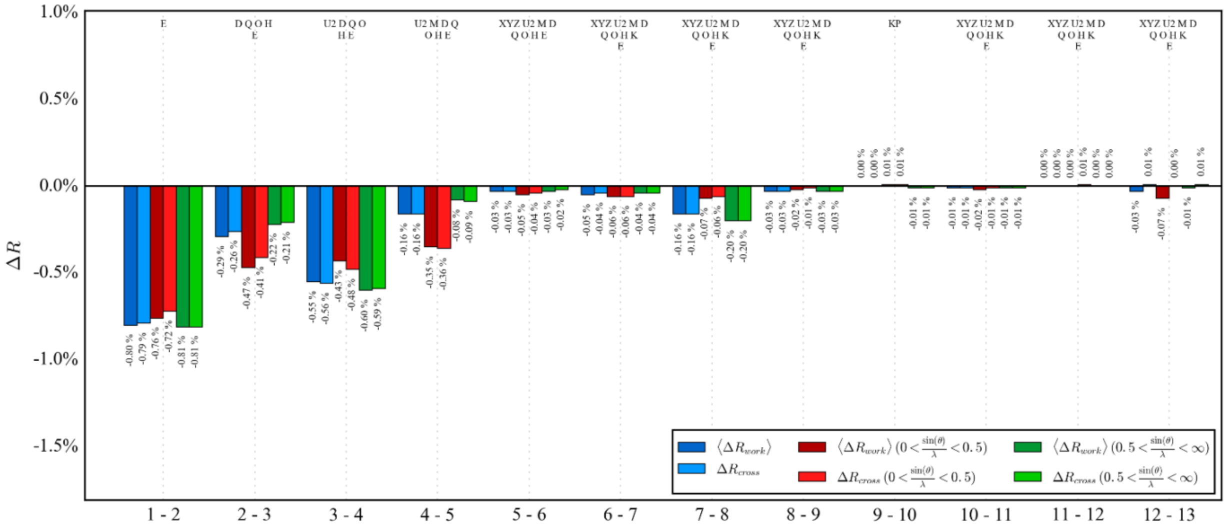

Figure S2-2. Rcross-Validation for experimental data.[1]

Table 2-4. Strategy for theoretical data. The final strategy goes up to and including step 6. The last two steps have been omitted as they lead to over-fitting. The last two steps have been omitted as they lead to over-fitting. See Figure S1-2. Abbreviations: **Sf:** scale factor; **MP:** multipoles; **M**: monopoles; **D**: dipoles; **Q**: quadrupoles; **O**: octupoles; **H**: hexadecapoles, **U2**: Uij, **k**: kappa, **k'**: kappa prime **noSym**: no local symmetry constraints, **GOF**. Goodness of Fit; **low-res data**: data < 0.5 sin(th)/l. Every new added parameter is marked in red.

Step	Refined Parameter	Data	MP-Param	Ratio low-res Data to MP, k & k'	Param	Ratio Data to Param	GOF	R(F²)
1	Sf	10838	0	0	1	10838	13.932	0.0154
2	Sf, DQOH	10838	58	5.8	59	183.7	5.901	0.0052
3	Sf, MDQOH	10838	64	5.2	65	169.3	4.793	0.0045
4	Sf, MDQOH, k	10838	64	5	67	161.8	4.379	0.0044
5	Sf, k'	10838	0	84	4	2709.5	4.204	0.0042
6	Sf, MDQOH, k	10838	64	5	68	161.8	4.048	0.0041
7	Sf, MDQOH, k, noCon [O]	10838	109	3	113	96.8	3.861	0.0041
8	Sf, MDQOH, k, m -> noSym [O]	10838	149	2.2	153	71.3	3.744	0.0040

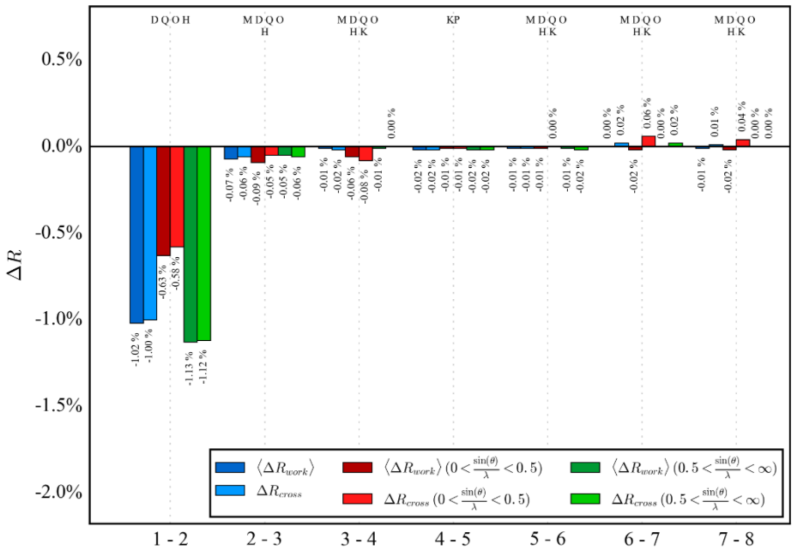

Figure S2-3. R_{cross}-Validation for DFT data.[1]

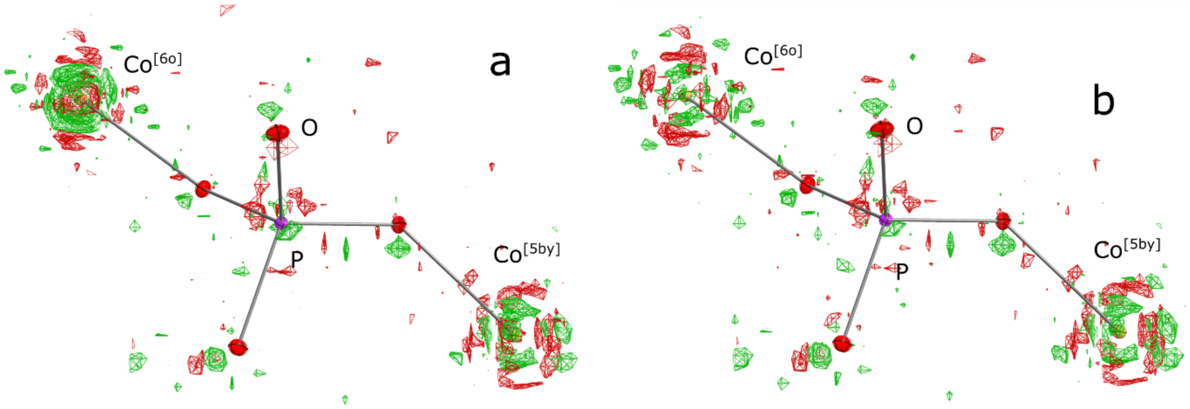

Figure S2-4. Residual density for the model a) without refining the second monopole and the model b) with refined second monopole. The isosurface value is ±0.26 eÅ^{-3}. Positive contours are plotted with green lines and negative contours are plotted with red lines

Figure S1-4 shows features of positive and negative residual density to be maintained around both cobalt atoms. However, the highest residual peak appears next to an oxygen atom in a distance of about 0.3 Å. The other three oxygen atoms have relatively high residual electron peaks at similar distances as well, indicating the multipolar model itself to be not good enough to accurately describe the electron density around the oxygen atoms. Different models that were tested, such as the inclusion of the anharmonic Gram-Chalier[2] coefficients, but could not significantly reduce the residual density. Since the refinement against the theoretical data also led to a similar result, we came to the conclusion that there were no errors in the experimental data. Several multipole models were tested to describe the electron density. The aim was to get the best model in terms of various quality criteria, such as residuals and R values. The best model is described here. Other tested models are discussed in the section below. Multipoles including the hexadecapoles were refined for all atoms except Co(6). For Co(6) only multipoles and other parameters were refined that fulfill the symmetry condition of an inversion center. The radial parameters κ and κ' were refined. To correct for extinction a parameter of type -1 with a Lorentzian mosaic distribution was refined. For the phosphorus and the oxygen atoms a non-crystallographic 3m and m symmetry was maintained, respectively. Suspending the symmetry would have led to a very adverse low-order data to multipole parameter ratio and overfitting, as proven by cross validation[1] (see SI). The 4s electrons of transition metals contribute very little to the total scattering. Hence only a few low order reflections contain information about 4s electrons which frequently are affected by absorption and extinction effects. Therefore, the general approach is to fix the 4s electrons in the core. In retrospect, removing 4s electrons from the refineable valence sphere gave a better refinement. Additionally, a second monopole was refined for both cobalt atoms. This process facilitates a better modelling of the density distribution of transition metals. Especially for Co(6) there has been a clear improvement in reducing the residual density (Figure S1-4).

S3. Data quality for Multipole Refinement

S3.1. Experimental data

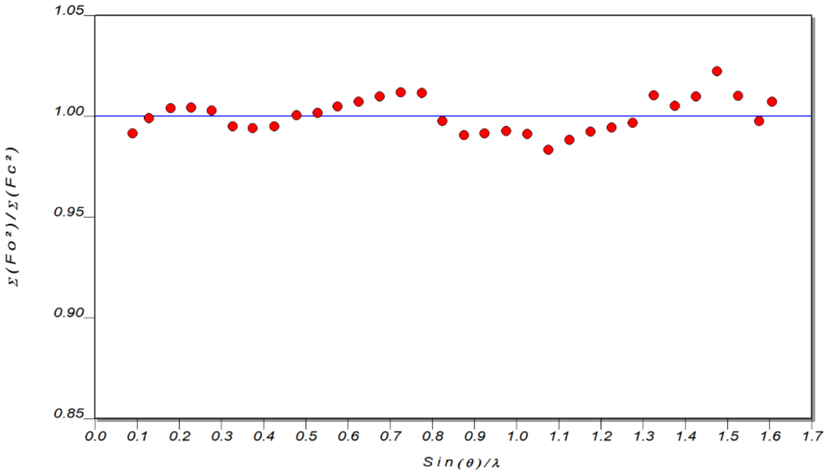

Figure S3-1. DRK Plot.[3,4]

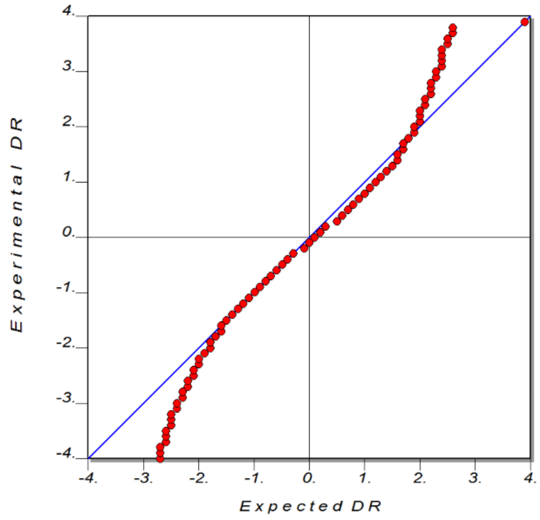

Figure S3-2. Normal Probability Plot.[3,5]

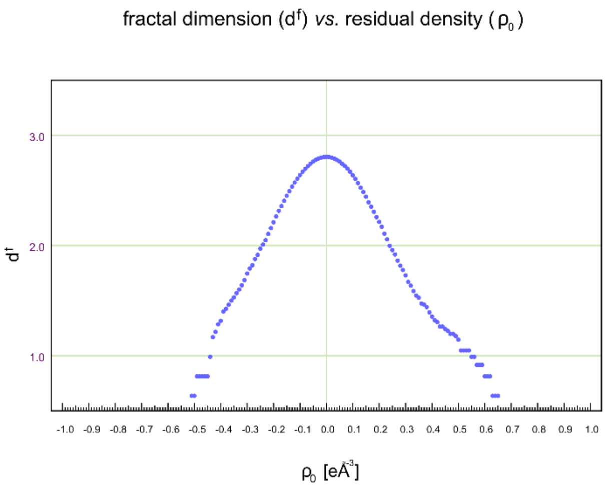

Figure S3-3. Henn-Meindl Plot.[6]

S3.2. DFT data

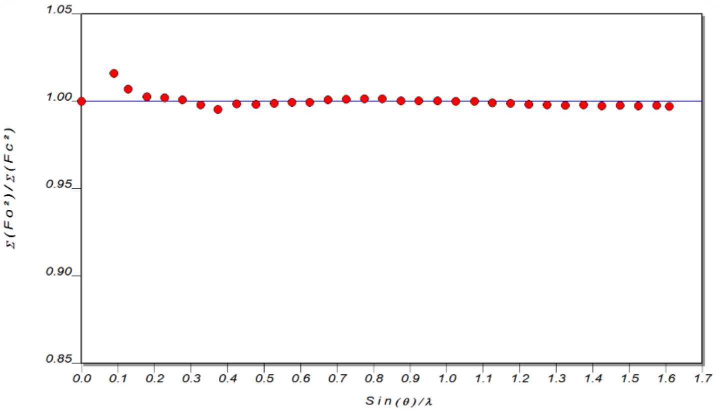

Figure S3-4. DRK Plot.[3,4]

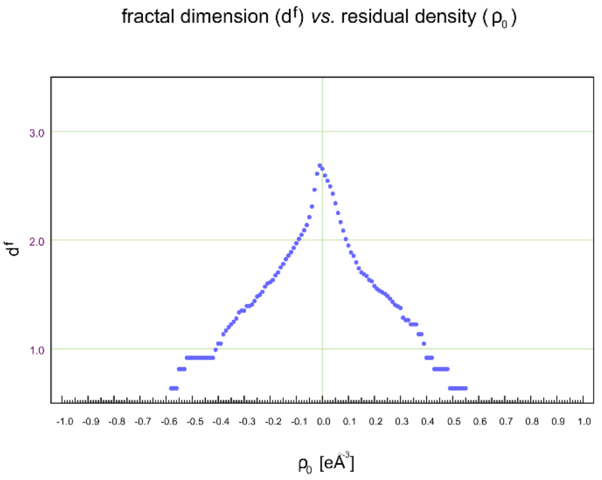

Figure S3-5. Henn-Meindl Plot. [6]

S4. Properties

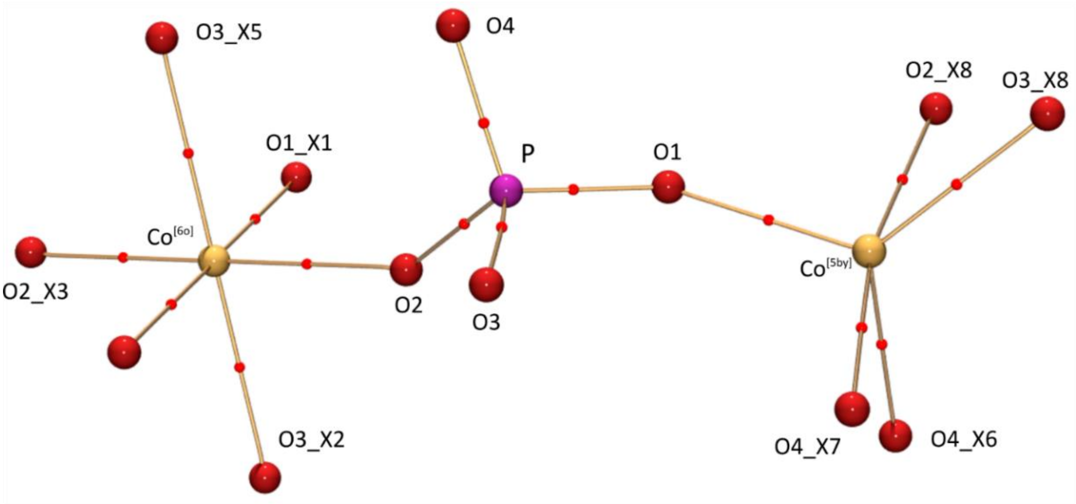

Figure S4-1. Structure of $Co_3(PO_4)_2$. The atoms are connected to each other via the bond path. The red points represent the BCPs. X1-X4 is a symmetry code. X1: -1+x, +y, +z; X2: -1/2+x, 1/2-y, -1/2+z; X3: -x, 1-y, 1-z; X4: 1-x, 1-y, 1-z; X5: 1/2-x, 1/2+y, 3/2-z; X6: 3/2-x, -1/2+y, 3/2-z; X7: 1/2+x, 1/2-y, -1/2+z; X8: 1+x, +y, +z.

Table S4-1. Calculated properties for MM-Expt (bold) and MM-DFT (normal). All errors shown below are determined by cross-validation[1].

A	*B*	ρ(r_{BCP}) ($eÅ^{-3}$)	∇^2ρ(r_{BCP}) ($eÅ^{-5}$)	R(*A-B*) (*Å*)	R(*A-BCP*) (*Å*)	R(*B-BCP*) (*Å*)	ε	*Hessian Eigenvalues*		
$Co^{[6o]}$	O2	**0.4060(9)**	**7.482(11)**	**2.05650(6)**	**1.0081(4)**	**1.0484(4)**	**0.050(6)**	**-1.78(8)**	**-1.69(8)**	**10.95(2)**
		0.4320(17)	7.320(15)	2.05630(15)	1.0270(6)	1.0294(7)	0.060(10)	-1.92(13)	-1.81(14)	11.04(3)
$Co^{[6o]}$	O1_X1	**0.3340(10)**	**5.659(12)**	**2.14380(5)**	**1.0464(5)**	**1.0974(5)**	**0.030(5)**	**-1.36(8)**	**-1.31(6)**	**8.33(2)**
		0.358(2)	5.662(15)	2.14370(2)	1.0662(14)	1.0776(14)	0.070(12)	-1.52(3)	-1.42(1)	8.60(6)
$Co^{[6o]}$	O3_X5	**0.3190(8)**	**5.256(13)**	**2.16940(7)**	**1.0555(7)**	**1.1139(7)**	**0.020(2)**	**-1.28(8)**	**-1.25(7)**	**7.78(2)**
		0.339(2)	5.171(17)	2.16940(2)	1.0780(15)	1.0914(15)	0.050(14)	-1.38(3)	-1.32(16)	7.87(5)
$Co^{[5by]}$	O1	**0.524(2)**	**9.94(2)**	**1.97740(6)**	**0.9731(5)**	**1.0043(5)**	**0.050(3)**	**-2.55(17)**	**-2.43(1)**	**14.91(5)**
		0.532(4)	9.60(3)	1.97720(4)	0.9850(12)	0.9922(12)	0.030(7)	-2.56(5)	-2.48(4)	14.64(8)
$Co^{[5by]}$	O2_X8	**0.2980(6)**	**4.384(9)**	**2.22180(7)**	**1.0942(6)**	**1.1276(6)**	**0.050(3)**	**-1.08(4)**	**-1.03(5)**	**6.500(18)**
		0.299(1)	4.307(18)	2.2217(0)	1.1118(12)	1.1099(12)	0.07(2)	-1.08(11)	-1.01(2)	6.39(4)
$Co^{[5by]}$	O3_X8	**0.4620(11)**	**8.434(13)**	**2.01900(6)**	**0.9995(2)**	**1.0195(3)**	**0.030(4)**	**-2.11(7)**	**-2.04(10)**	**12.59(2)**
		0.467(2)	8.22(2)	2.01890(9)	1.0094(9)	1.0095(9)	0.040(7)	-2.09(13)	-2.01(2)	12.32(3)
$Co^{[5by]}$	O4_X6	**0.5070(13)**	**9.639(17)**	**1.98890(6)**	**0.9813(6)**	**1.0076(6)**	**0.040(3)**	**-2.47(15)**	**-2.38(16)**	**14.49(4)**
		0.515(4)	9.36(4)	1.98880(4)	0.9942(12)	0.9946(13)	0.020(7)	-2.49(5)	-2.44(4)	14.28(8)
$Co^{[5by]}$	O4_X7	**0.4810(9)**	**8.980(13)**	**2.00790(8)**	**0.9937(3)**	**1.0143(4)**	**0.020(4)**	**-2.25(5)**	**-2.21(12)**	**13.44(2)**

		0.490(3)	8.75(2)	2.00800(2)	1.0033(11)	1.0047(11)	0.010(10)	-2.3(3)	-2.28(3)	13.33(6)
P1	O1	**1.587(7)**	**4.8(3)**	**1.53340(7)**	**0.6332(4)**	**0.9002(3)**	**0.030(8)**	**-13.43(13)**	**-13.09(7)**	**31.3(2)**
		1.583(7)	2.6(6)	1.53330(6)	0.6431(12)	0.8902(12)	0.010(5)	-12.19(13)	-12.06(12)	26.8(6)
P1	O2	**1.606(14)**	**5.0(6)**	**1.53180(6)**	**0.6327(3)**	**0.8991(3)**	**0.01(0)**	**-13.3(1)**	**-13.2(2)**	**31.5(3)**
		1.610(14)	2.9(8)	1.53170(3)	0.6423(12)	0.8895(12)	0.0(0)	-12.1(2)	-12.1(2)	27.0(6)
P1	O3	**1.539(7)**	**1.9(3)**	**1.55560(13)**	**0.6416(4)**	**0.9140(5)**	**0.020(4)**	**-12.61(11)**	**-12.36(7)**	**26.9(2)**
		1.537(7)	-0.1(6)	1.5555(2)	0.6527(15)	0.9028(13)	0.010(6)	-11.52(12)	-11.37(11)	22.8(6)
P1	O4	**1.569(7)**	**3.7(3)**	**1.54130(6)**	**0.6362(4)**	**0.9051(4)**	**0.010(6)**	**-13.08(12)**	**-12.91(8)**	**29.7(2)**
		1.564(7)	1.5(6)	1.54140(7)	0.6466(13)	0.8948(13)	0.010(7)	-11.95(14)	-11.85(11)	25.3(6)

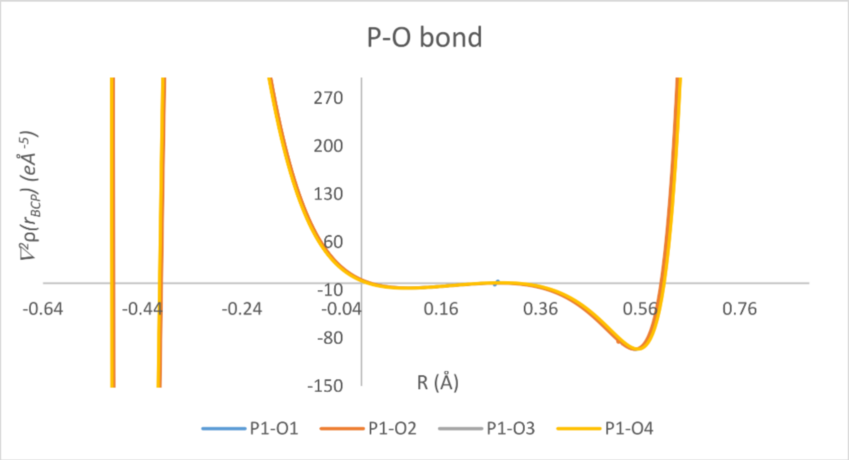

Figure S4-2. Laplacian profile along the four P-O bondpaths.

Table S4-2. Calculated Energy densities for MM-Expt (bold) and MM-DFT (normal).

A	*B*	G(r_{bcp}) (E_h Å^{-3})	G(r_{BCP})/ ρ(r_{BCP}) (E_h/e)	V(r_{BCP}) (E_h Å^{-3})	|V|(r_{BCP}) /G(r_{BCP})	H(r_{BCP}) (E_h Å^{-3})	H(r_{BCP})/ ρ(r_{BCP}) (E_h/e)
Co[6o]	O2	**0.53**	**1.30**	**-0.53**	**1.00**	**0.00**	**0.00**
		0.54	1.25	-0.57	1.06	-0.03	-0.07
Co[6o]	O1_X1	**0.39**	**1.18**	**-0.39**	**1.00**	**0.00**	**0.00**
		0.41	1.14	-0.42	1.02	-0.01	-0.03
Co[6o]	O3_X5	**0.37**	**1.14**	**-0.36**	**0.97**	**0.00**	**0.00**
		0.37	1.10	-0.39	1.05	-0.01	-0.03
Co[5by]	O1	**0.74**	**1.41**	**-0.78**	**1.05**	**-0.04**	**-0.08**
		0.73	1.37	-0.79	1.08	-0.06	-0.11
Co[5by]	O2_X8	**0.31**	**1.04**	**-0.32**	**1.03**	**0.00**	**0.00**
		0.31	1.03	-0.32	1.03	-0.01	-0.03
Co[5by]	O3_X8	**0.62**	**1.33**	**-0.64**	**1.03**	**-0.03**	**-0.07**
		0.61	1.31	-0.64	1.05	-0.03	-0.06
Co[5by]	O4_X6	**0.71**	**1.40**	**-0.74**	**1.04**	**-0.03**	**-0.06**
		0.70	1.36	-0.75	1.07	-0.05	-0.10
Co[5by]	O4_X7	**0.66**	**1.37**	**-0.68**	**1.03**	**-0.03**	**-0.06**
		0.65	1.33	-0.69	1.06	-0.04	-0.08
P1	O1	**1.96**	**1.24**	**-3.59**	**1.83**	**-1.62**	**-1.02**
		1.85	1.17	-3.52	1.90	-1.67	-1.06
P1	O2	**2.01**	**1.25**	**-3.66**	**1.82**	**-1.65**	**-1.02**
		1.91	1.19	-3.62	1.90	-1.71	-1.06
P1	O3	**1.74**	**1.13**	**-3.35**	**1.93**	**-1.61**	**-1.05**
		1.64	1.07	-3.29	2.01	-1.65	-1.07
P1	O4	**1.88**	**1.20**	**-3.49**	**1.86**	**-1.62**	**-1.03**
		1.77	1.13	-3.43	1.94	-1.66	-1.06

Table S4-3. Valence shell charge concentrations and depletions.

VSCCs		Experiment				Theory		
$Co^{[6o]}$		$\rho(r_{BCP})$ (eÅ $^{-3}$)	$\nabla^2\rho(r_{BCP})$ (eÅ $^{-5}$)	Distance (Å)		$\rho(r_{BCP})$ (eÅ $^{-3}$)	$\nabla^2\rho(r_{BCP})$ (eÅ $^{-5}$)	Distance (Å)
	1	29.6	-1424	0.31		32.6	-1859	0.30
	2	29.2	-1367	0.31		32.7	-1882	0.30
	3	31.0	-1737	0.30		32.7	-1861	0.30
$Co^{[5by]}$	1	32.3	-1889	0.30		32.9	-1889	0.30
	2	31.7	-1817	0.30		32.6	-1860	0.30
	3	32.3	-1890	0.30		32.8	-1878	0.30
	4	32.1	-1862	0.30		32.8	-1878	0.30
	5	31.5	-1793	0.30		32.5	-1859	0.30
	6	32.6	-1916	0.30		32.8	-1888	0.30

Charge depletions		$\rho(r_{BCP})$ (eÅ $^{-3}$)	$\nabla^2\rho(r_{BCP})$ (eÅ $^{-5}$)	Distance (Å)		$\rho(r_{BCP})$ (eÅ $^{-3}$)	$\nabla^2\rho(r_{BCP})$ (eÅ $^{-5}$)	Distance (Å)
$Co^{[6o]}$	1	8.4	264	0.48		9.0	293	0.47
	2	8.4	261	0.48		9.3	302	0.47
	3	8.5	268	0.48		9.5	316	0.46
	4	-	-	-		9.0	293	0.47
$Co^{[5by]}$	1	8.5	264	0.48		9.1	293	0.47
	2	8.8	286	0.47		9.1	297	0.47
	3	9.1	299	0.46		9.4	303	0.47
	4	8.9	286	0.46		9.4	311	0.46
	5	9.0	288	0.47		9.3	312	0.46
	6	8.8	285	0.47		9.4	308	0.46
	7	9.2	301	0.46		9.1	299	0.46
	8	8.5	262	0.48		9.0	293	0.47

Table S4-4. Bader charges for MM-Expt and MM-DFT.

atom	MM-Expt	MM-DFT
$Co^{[6o]}$	1.98	1.22
$Co^{[5by]}$	1.59	1.11
P1	3.94	3.69
O1	-1.62	-1.33
O2	-1.62	-1.36
O3	-1.61	-1.33
O4	-1.66	-1.38

S5. Plots for comparison of MM-Expt with MM-DFT Refinement.

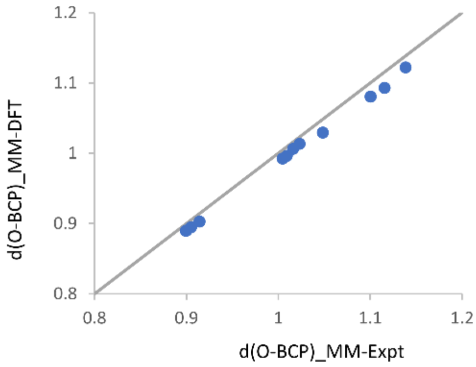

Figure S5-1. Distances of the BCP from the oxygen atom position from theory above those from the experiment.

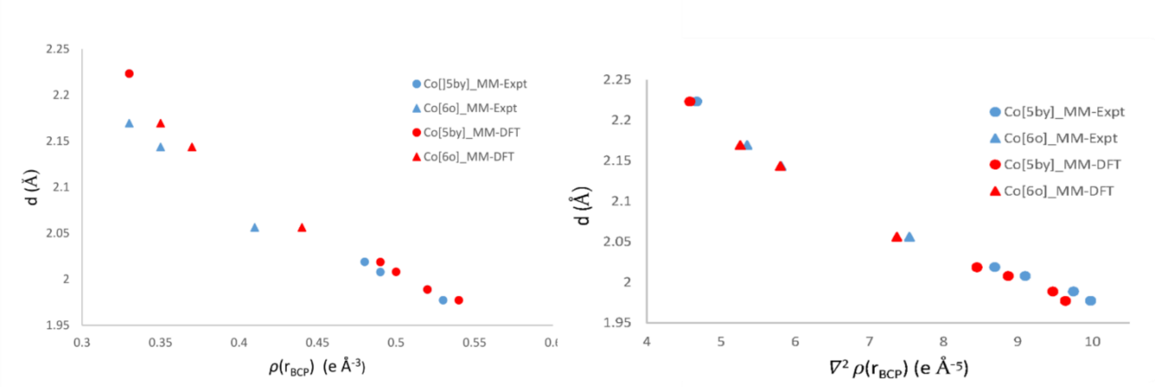

Figure S5-2. Experimental Co–O bond lengths plotted with respect to a) $\rho(r_{BCP})$ and b) $\nabla^2\rho(r_{BCP})$ values comparing the experimental (blue) and theoretical (red) data.

S6. Maximally localized Wannier functions.

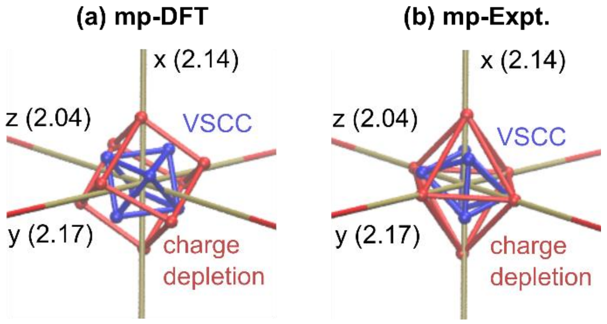

Figure S6-1. Positions of the VSCCs (blue) and charge depletions (red) around the six-coordinated Co ion in cobalt orthophosphate as calculated from multipole refinements from (a) DFT data, and (b) x-ray diffraction data (experimental).

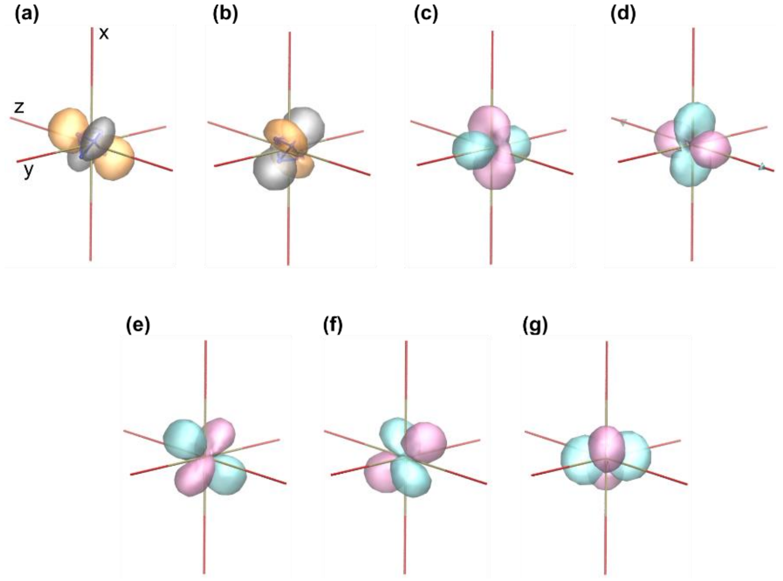

Figure S6-2. Maximally localized Wannier functions for Co(6) (isovalue ± 0.2 eÅ^{-3}), calculated from occupied bands in a DFT calculation. Orange/gray: spin-down orbitals, pink/cyan: spinup orbitals. The chosen local coordinate system is given in (a). In (a-b), the VSCCs from Figure S5-1 are also shown.

S7. Non-periodic DFT calculations

During the multipole refinement for fitting the electron density, it is necessary to first assign a local coordinate system to the atoms, with predetermined spatial directions for the spherical harmonic functions describing dipoles, quadrupoles, etc. Because the actual coordination polyhedra around the Co ions in $Co_3(PO_4)_2$ are distorted from the ideal structures, we used DFT calculations, together with manual judgment, to determine the pertinent local coordinate systems. The first coordination sphere, including the entire PO_4^{3-} units, around the two types of Co ions were cut out from the bulk crystal, giving a $[Co^{II}(OPO_3)_4]^{10-}$ complex for the five-fold coordinated Co ion (for which one of the PO_4^{3-} ligands is bidentate), and a $[Co^{II}(OPO_3)_6]^{16-}$ complex for the six-coordinated Co ion. The all-electron code ADF[7] was used to perform single-point calculations at the B3LYP level of theory with a double zeta plus polarization (DZP) basis set. Because the goal of these calculations was to identify suitable coordinate systems for the multipole refinement, we elected to perform spin-restricted calculations, forcing the spin-up and spin-down electrons for the doubly occupied orbitals to occupy exactly the same spatial part. We found that the coordinate system assignment from spin-unrestricted calculations (not shown here) was more ambiguous, as the spin-up and spin-down electrons did not occupy the same spatial parts, and also did not have the characteristic d orbital "shapes".

However, we note that the occupation numbers of the orbitals depended on whether spin-restricted or spin-unrestricted calculations were performed; in the periodic calculations for the bulk crystal described in the main text, we employed spin-unrestricted calculations to obtain the best possible description of the electron density, which resulted in different occupation numbers compared to those presented in this section. Figure S6-1 shows the calculated spin-restricted Kohn-Sham orbitals for the two different Co complexes. For the five-fold coordinated Co ion, the lobes of the (in this case unoccupied) d_{z2} orbital pointed approximately in the direction of the axial ligands, as would be expected from crystal field theory for a trigonal bipyramidal complex. The x direction was determined to be approximately directed towards the equatorial ligand on one of the monodentate PO_4^{3-}(Co-O distance: 2.008 Å) (Table 1), as judged from the lobes on the doubly occupied d_{xz}, d_{yz}, d_{xy}, and the singly occupied $d_{x^2-y^2}$ orbitals. For the Co(6) ion, the lobes of the unoccupied dz^2 orbital pointed towards the nearest ligands (Co–O distance: 2.056 Å), and the x direction was arbitrarily assigned to point approximately towards the second-nearest ligand (Co–O distance: 2.144 Å).

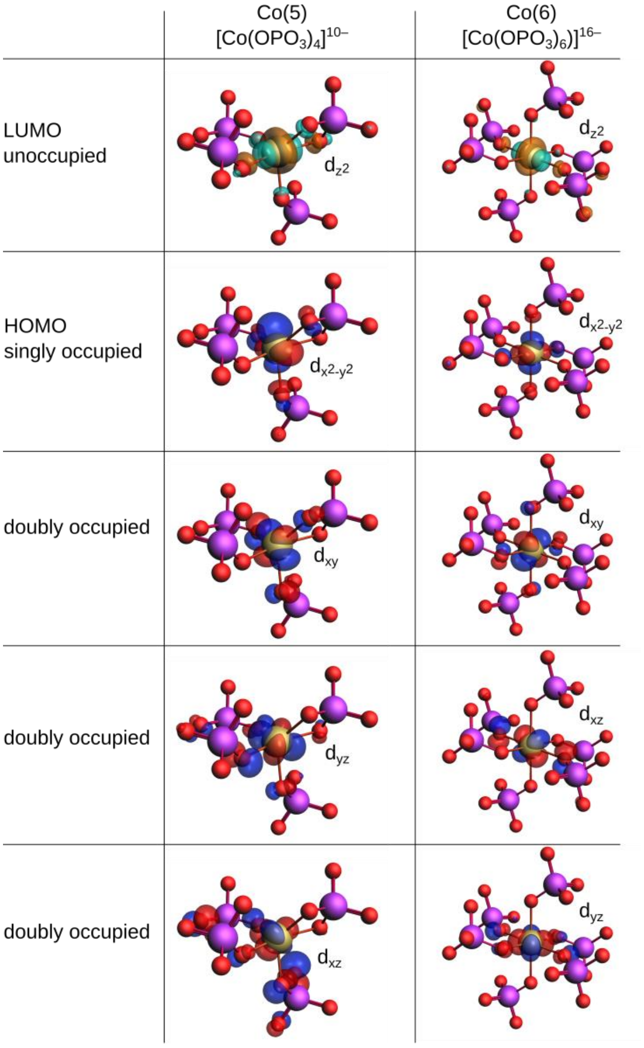

Figure S7-1. Kohn-Sham orbitals from restricted hybrid DFT calculations on complexes cut out from the cobalt orthophosphate crystal. The labels indicate the primary contributing d orbital to the molecular orbital. The iso-values are ±0.05 bohr^{-3}.

This coordinate system assignment was used to (i) perform the multipole refinement, and (ii) to estimate the d orbital populations, from both the multipole refinement as well as directly from the periodic electronic structure calculations. However, we emphasize that the quality of the multipole refinement, as evaluated by the residual error, was largely independent of the chosen local coordinate system. The choice of the local coordinate system only had noticeable impact on the calculated d orbital populations, which serves as useful validation of the simulation protocol and local coordinate system assignment. However, as discussed earlier, the usefulness of calculating the d orbital populations decreases when the Co ions have very distorted coordination polyhedra, and when there are several different electronic configurations that are almost degenerate. Indeed, the self-consistent optimization of the Kohn-Shan orbitals in the all-electron DFT calculations on the Co complexes in Figure S6-1, especially for the six-coordinated Co ion, converged very slowly, suggesting the presence of several states close in energy. We emphasize that the role of the all-electron DFT calculations described in this section was very small. The periodic, spin-polarized, calculations in the main text played a much more important role.

S8. Side views of the crystal planes

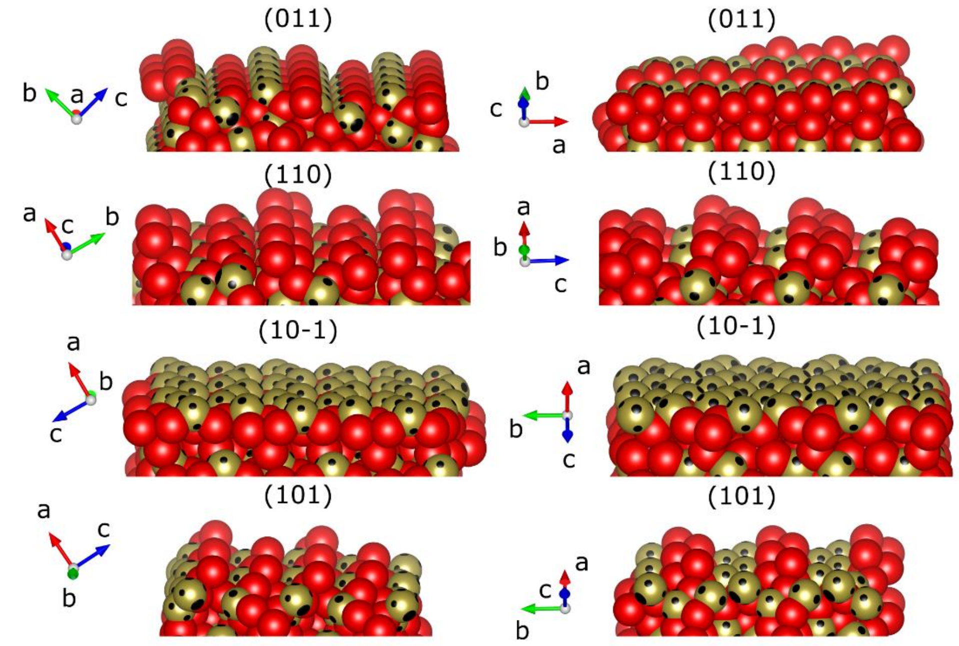

S9. Plane-wave bulk and surface DFT calculations

To calculate the relative stability of the high-spin and low-spin configurations, as well as the adsorption energies of butan-2-ol on different surface sites, spin-polarized PBE+U (U = 3 eV) calculations using ultrasoft pseudopotentials[8] and a plane-wave basis set with energy cutoff 400 eV were performed using Quantum ESPRESSO[9]. Calculations were performed for (i) the bulk crystal conventional unit cell, (ii) a (011) surface slab with two different terminations, and (iii) an isolated butan-2-ol molecule. For the bulk conventional unit cell, a 5 × 3 × 3 k-point grid was used, and for the other calculations only the Γ point was used. The slab was roughly 11 Å thick and the vacuum gap along the surface normal direction 14 Å; a (2 × 1) surface supercell was used, such that the resulting simulation box had dimensions a = 10.139 Å, b = 11.257 Å, c = 25.000 Å, α = 90.000°, β = 90.000°, γ = 87.234°. The "top" and "bottom" sides of the slabs had two different terminations in order to ensure that the slab as a whole had the $Co_3(PO_4)_2$ stoichiometry.

We found the high-spin solution to be 0.83 eV per Co atom more stable than the low-spin solution for the bulk crystal.

For the adsorption energy calculations, we placed a butan-2-ol molecule at seven different reasonable initial positions (judged by manual inspection) on the surface and relaxed the positions of all atoms. Because there are multiple local minima on the potential energy surfaces for butan-2-ol itself, as well as when it is adsorbed on the surface, it is possible that we did not find the global minimum for the adsorbed structure (which is always the case in these types of calculations for any non-trivial adsorbate and/or surface).

Figure S9-1 shows the most stable adsorption configurations found at several different adsorption sites. The greatest adsorption energy (i.e., the most favourable adsorption, Site 3 in Figure S9-1) was calculated for butan-2-ol adsorbed on a Co atom corresponding to one of the two Co[5by] atoms in the bulk crystal being 3.032 Å apart, as also suggested by the electron density (ED) analysis in the main text. Thus, the present adsorption energy calculations confirm that the reasoning based on ED in the main text is appropriate. However, as can be seen in Figure S9-1, the next-most stable adsorption site is only 0.02 eV less favourable, suggesting the possibility that butan-2-ol may adsorb on several different sites close in energy. This second most stable adsorption site is onto a Co atom that, after surface relaxation, is also very close to another Co atom (3.07 Å). Thus, in general, it appears that neighbouring Co atoms provide good adsorption sites for butan-2-ol. How such possibilities are affected by surface relaxation, as well as a more quantitatively accurate evaluation of the adsorption energies, would be interesting avenues to explore in more detail in future work.

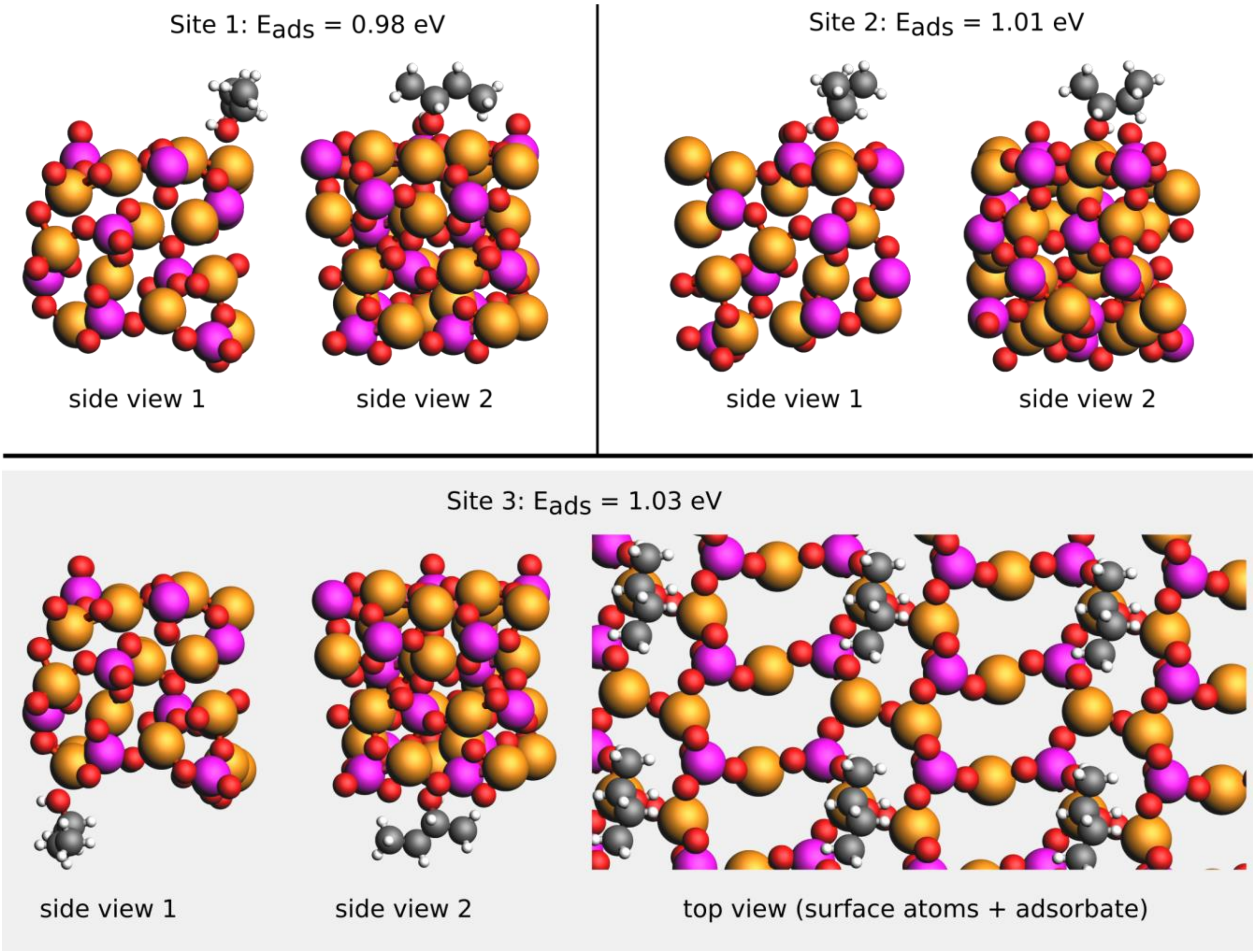

Figure S9-1. The three most stable adsorption sites found for butan-2-ol on the (011) face of $Co_3(PO_4)_2$. Side views 1 and 2 correspond to the left and right sides of the (011) view in Figure S8. The most stable adsorption site, Site 3, is highlighted with a gray background. The side views show one whole simulation supercell. The top view for site 3 shows several periodically repeated supercells.

[1] L. Krause, B. Niepötter, C. J. Schürmann, D. Stalke, R. Herbst-Irmer *IUCrJ* **2017**, *4*, 420.

[2] R. Herbst-Irmer, J. Henn, J. J. Holstein, C. B. Hübschle, B. Dittrich, D. Stern, D. Kratzert, D. Stalke *J. Phys. Chem. A* **2013**, *117*, 633.

[3] V. V. Zhurov, E. A. Zhurova, A. A. Pinkerton *J. Appl. Crystallogr.* **2008**, *41*, 340.

[4] A. Stash, *DRKplot*, Moscow, **2007**.

[5] S. C. Abrahams, E. T. Keve, *Acta Crystallogr. A* **1971**, *27*, 157.

[6] K. Meindl, J. Henn, *Acta Crystallogr. A* **2008**, *64*, 404.

[7] a) Baerends, E. J. et al. *ADF2017*, SCM, Theoretical Chemistry, Vrije Universiteit,, Amsterdam; b) C. Fonseca Guerra, J. G. Snijders, G. te Velde, E. J. Baerends, *Theor. Chem. Acc.* **1998**, *99*, 391; c) G. te Velde, F. M. Bickelhaupt, E. J. Baerends, C. Fonseca Guerra, S. J. A. van Gisbergen, J. G. Snijders, T. Ziegler *J. Comput. Chem.* **2001**, *22*, 931.

[8] K. F. Garritya, J. W. Bennetta, K. M. Rabea, D. Vanderbilt *Comp. Mat. Sci.* **2014**, *81*, 1.

[9] a) P. Giannozzi, et al. *J. Phys.: Condens. Matter* **2017**, *29*, 465901; b) P. Giannozzi, et al. *J. Phys.: Condens. Matter* **2009**, *21*, 395502.
